# Is it time to align adolescent diets with the Planetary Health Diet? An observational study on early cardiovascular health

**DOI:** 10.3389/fnut.2025.1739577

**Published:** 2026-02-03

**Authors:** David Murcia-Lesmes, Emily P. Laveriano-Santos, Ramón Estruch, Marina Corrado, Camila Arancibia-Riveros, Ana María Ruiz-León, Rosa Casas, Miguel Camafort, Jesús Martínez-Gómez, Amaya de Cos-Gandoy, Patricia Bodega, Gloria Santos-Beneit, Juan M. Fernández-Alvira, Rodrigo Fernández-Jiménez, Rosa M. Lamuela-Raventós, Sara Castro-Barquero

**Affiliations:** 1 Polyphenol Research Group, Departament de Nutrició, Ciències de l’Alimentació i Gastronomía, Facultat de Farmàcia i Ciències de l’Alimentació, Barcelona, Spain; 2 Institut de Nutrició i Seguretat Alimentària (INSA-UB), Universitat de Barcelona, Barcelona, Spain; 3 Barcelona Institute for Global Health (ISGlobal), Barcelona, Spain; 4 Centro de Investigación Biomédica en Red de Fisiopatología de la Obesidad y Nutrición (CIBEROBN), Instituto de Salud Carlos III, Madrid, Spain; 5 Department of Internal Medicine, Institut d’Investigacions Biomèdiques August Pi Sunyer (IDIBAPS), Hospital Clinic, University of Barcelona, Barcelona, Spain; 6 Centro Nacional de Investigaciones Cardiovasculares (CNIC), Madrid, Spain; 7 Foundation for Science, Health and Education (SHE Foundation), Barcelona, Spain; 8 CIBER de Enfermedades Cardiovasculares (CIBERCV), Madrid, Spain; 9 Hospital Universitario Clínico San Carlos, IdISSC, Madrid, Spain

**Keywords:** adolescents, cardiovascular health, nutritional epidemiology, Planetary Diet, plant-based diet, prevention, prospective study, sustainability

## Abstract

**Background:**

As the impact of early adoption of a sustainable plant-based diet on cardiometabolic biomarkers remains unexplored, we assessed whether they are associated with the Planetary Health Diet Index (PHDI) in adolescents.

**Methods:**

This prospective study was conducted within the SI! Program for Secondary Schools trial (SI! Program) in 886 adolescents (12 years ± 0.4 at cohort entry; 49.1% female) followed during 4 years in Spain. The PHDI scores were derived from validated food frequency questionnaires. Multivariable-adjusted Cox proportional-hazards models (HRs) analyzed the association between PHDI and risk of new-onset high blood pressure (BP), obesity, and elevated plasma cardiometabolic biomarkers. Additionally, mixed models assessed changes in those parameters.

**Results:**

High adherence to the PHDI_(Q4 vs. Q1)_ is associated with a reduced risk of high BP by 81% (HR: 0.19 [95% CI: 0.11, 0.34]), plasma glucose by 47% (HR: 0.53 [95% CI: 0.48, 0.58]), triglycerides (TG) by 66% (HR: 0.34 [95% CI: 0.18, 0.65]), total cholesterol by 51% (HR: 0.49 [95% CI: 0.34, 0.69]), and non-high density lipoprotein cholesterol (non-HDL-C) by 74% (HR: 0.26 [95% CI: 0.13, 0.50]) in Cox models. Mixed models show inverse associations with higher PHDI and blood glucose (−5.23 mg/dL [95% CI: −10.35, −0.10]), TG (−2.48 mg/dL [95% CI: −3.65, −1.30]), and body mass index (BMI) z-score (−0.02 [95% CI: −0.03, 0.00]).

**Conclusion:**

This study stands out as greater adherence to the PHDI is inversely associated with cardiometabolic biomarkers in adolescents, highlighting nutritional benefits of the Planetary Health Diet and its role in preventing the development of cardiovascular diseases and early detection.

## 1 Introduction

Food consumption impacts both human health and environmental resources, making the adoption of sustainable consumption practices essential for preserving future food production (1). Agriculture accounts for approximately 25% of total greenhouse gas emissions, occupies around 40% of the Earth’s surface, and consumes 70% of global freshwater resources (2). Thus, the global food system is surpassing several planetary boundaries, with its stability increasingly threatened by ecosystem overexploitation and pollution. Dietary changes aimed at fostering sustainable eating habits can significantly reduce the demand for food items with a high carbon footprint, which pose a threat to the environment (1, 2). Those foods include red meat, processed meat, ultra-processed foods, sugar, and refined grains, all of which offer low nutritional benefits, due to their high content of saturated fats, cholesterol, sodium, added sugars, and refined starches (2).

The Planetary Diet, a healthy and sustainable diet, aligns with the achievement of Sustainable Development Goals by encouraging the consumption of nutrient-rich foods such as vegetables, fruits, whole grains, legumes, nuts, and unsaturated fats (3). This dietary pattern prioritizes the inclusion of plant-based foods, which are rich sources of dietary fiber, antioxidant bioactive compounds, including (poly) phenols and carotenoids, and vitamins (provitamin A, C, E); all of them known for health-promoting properties (4, 5). Numerous studies in adults have explored the adherence to the Planetary Health Diet and its association with cardiovascular diseases (6–10), cardiovascular events (11), and mortality (8, 12–14), demonstrating promising benefits across these areas.

Most existing studies have focused on adult populations, limiting our understanding of the nutritional benefits of adopting the Planetary Health Diet at a younger age. Elevated biomarkers of cardiometabolic risk, such as lipid profile, blood pressure (BP), and plasma glucose, are known factors for cardiovascular disease in adolescents (15, 16) and cardiovascular events later in life (17). Therefore, the aim of this prospective cohort study is to assess the association between adherence to the Planetary Health Diet, measured by the Planetary Health Dietary Index (PHDI), and the risk of new-onset high blood pressure, obesity, and other elevated cardiometabolic risk factors during 4 years of follow-up in adolescents in Spain. Thus, we studied risk factors including obesity, high plasma glucose, low-density lipoprotein cholesterol (LDL-C), triglycerides (TG), total cholesterol, high-density lipoprotein cholesterol (HDL-C), and non-HDL-C. Additionally, we examined the relationship between changes in these cardiometabolic parameters, and systolic and diastolic blood pressures (SBP and DBP), body mass index (BMI) z-score, and waist-to-height ratio (WHtR).

## 2 Methods

### 2.1 Study population

The SI! Program for Secondary Schools trial is a cluster randomized controlled trial, which aimed to assess the effect of a lifestyle program on cardiovascular health among adolescents between 12 and 16 years, conducted in Spain (Metropolitan areas of Madrid and Barcelona) from 2017 to 2021 (https://fundacionshe.org/programa-si/). The intervention consisted of a comprehensive education program with short- and long-term interventions (2 and 4 years, respectively) and a standard curriculum (control). Participant selection considered students registered in the first year of secondary school at the engaged institutions. The study included 24 public secondary schools (17 in Barcelona and 7 in Madrid), encompassing 1,326 adolescents. The details of the study design and methodology can be found elsewhere (18). The SI! Program was registered at https://clinicaltrials.gov/ (NCT03504059) and adheres to the ethical standards outlined in the Declaration of Helsinki. The study was approved by the Committee for Ethical Research (CEI) of the Instituto de Salud Carlos III in Madrid (CEI PI 35/2016), the CEI of the Fundació Unió Catalana d’Hospitals (CEI 16/41), and the Bioethics Committee of the University of Barcelona (IRB00003099). All participants and their parents/legal guardians gave their written informed consent.

**Figure 1:**
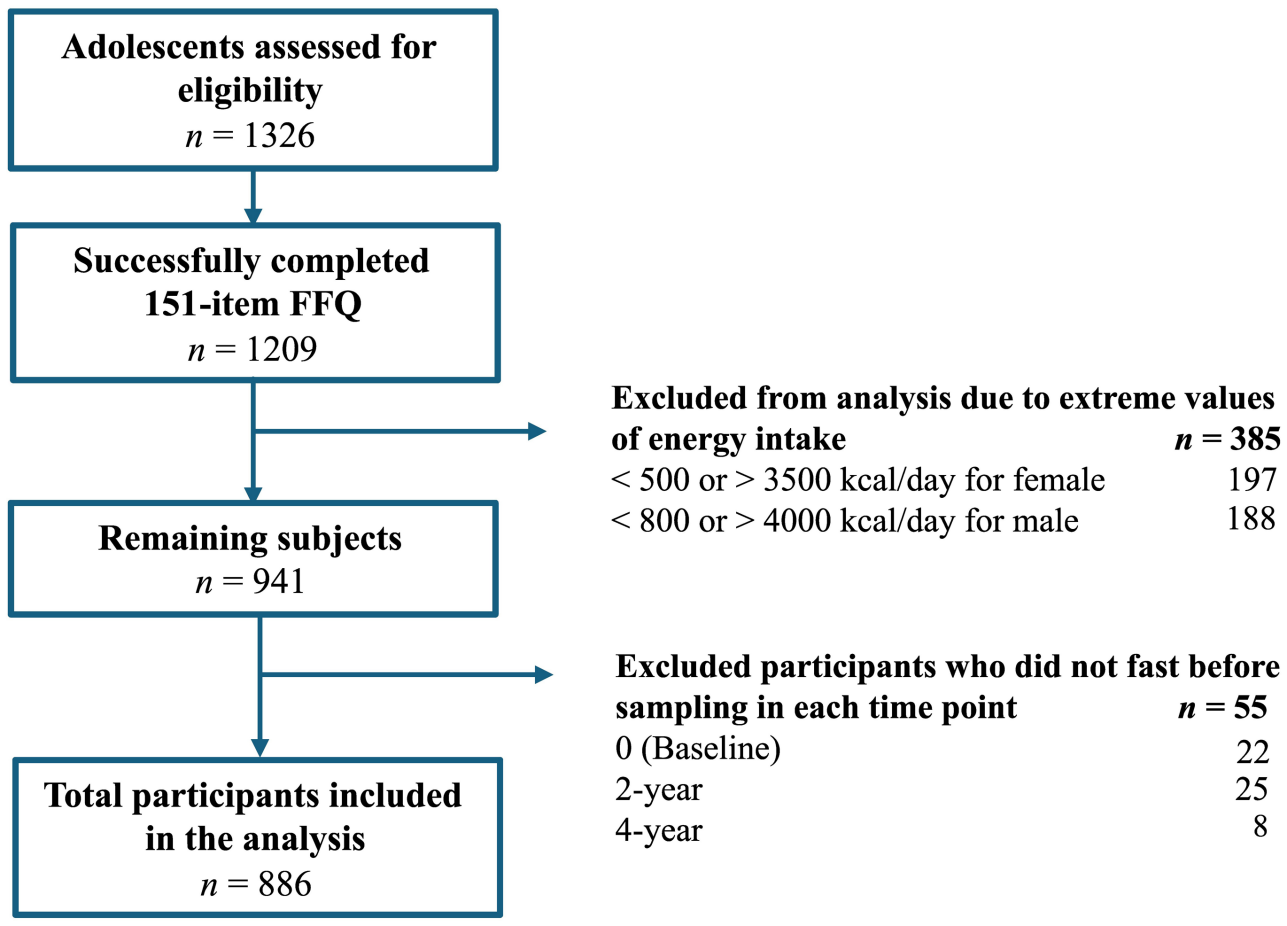
Flowchart of adolescents in the SI! Program included in the present study, *n* = 886.

The present prospective analysis incorporates data from baseline, 2 years, and 4 years of follow-up, focusing on the PHDI (exposure). Extreme values of total energy (<500 or >3,500 kcal/d for female and < 800 or > 4,000 kcal/d for male) (19) and non-fasting participants were removed from this analysis. Of the 1,326 adolescents who were randomly assigned, 886 participants were included in the final analysis (Figure 1).

### 2.2 Dietary and covariate assessment

Parents/caregivers completed online questionnaires regarding sociodemographic, lifestyle, and dietary factors. Parental education was derived from the highest level of education attained; if only one parent’s level was available, that level was used. Eating habits of adolescent students were reported by their parents/caregivers following the instructions of the research team and subsequently checked by trained dietitians. Eating pattern was assessed using a validated 151-item semi-quantitative Food Frequency Questionnaire (FFQ) (20). Food consumption, derived from this FFQ, was translated into energy and nutrient intake using Spanish food composition tables (21, 22). Meanwhile, adolescents filled out questionnaires regarding puberty development using pictograms (Stage I defined as prepubertal and Stage V defined as mature), and personnel performed standardized clinical measurements in the students’ school settings during school hours. Physical activity and sleep of adolescents were monitored through an Actigraph wGT3X-BT wearable accelerometer during 7 consecutive days.

### 2.3 Planetary Health Dietary Index (PHDI)

Adherence to the Planetary Health Diet was based on the methodology outlined by Bui et al. (12). The PHDI considers 15 recommendations based on predetermined cutoffs for each item promoting the consumption of whole grains, vegetables, (excluding starchy vegetables), fruits, legumes (including peanuts, pulses, or soy), and unsaturated fats, while encouraging a reduction in animal-based food sources (e.g., beef, lamb, pork, or chicken). It also advises moderation in saturated fats, refined grains, sugars, and added sugars. The highest score possible for each food group was 10, except for non-soy legumes and soy foods (a maximum of five points for each item). Since the diet was proposed over a specific caloric requirement, we standardized the diets to 2,500 kcal/day to meet the criteria. Participants’ scores were assigned proportionally between the maximum and minimum thresholds, and the score was calculated by summing the components. Hence, leading to potential scores ranging from 0 to 140, with higher scores indicating greater adherence to the PHDI. The cumulative average of the PHDI was used to reduce measurement errors during follow-up. Then, the PHDI score was divided into quartiles based on the distribution among participants: Q1: < 80.5 points; Q2: 80.5–90 points; Q3: 90.1–98.5 points; and Q4: > 98.5 points.

### 2.4 Cardiometabolic parameters measurement

BP was measured using an OMRON M6 monitor, with readings taken twice at 2–3-min intervals. If variation exceeded >10 mmHg for systolic SBP or >5 mmHg for DBP, additional measurements were taken. Body weight and height were measured using calibrated electronic scales (OMRON BF511) and portable stadiometers (Seca 213), respectively. BMI was calculated by dividing adolescent body weight in kilograms by the square of his height in meters. Waist circumference was measured in triplicate to the nearest 0.1 cm using a non-stretchable Holtain tape, and the WHtR was calculated by dividing waist circumference by height in centimeters. Fasting blood glucose, LDL-C, TG, total cholesterol, and HDL-C levels were assessed using a CardioCheck Plus device (Polymer Technology System Inc.) and PTS-Panels test strips on capillary blood.

### 2.5 Outcome ascertainment and thresholds

The primary outcomes in this study included risk of new-onset high BP based on age, sex, and height according to the American Academy of Pediatrics (*for adolescents <13 years: ≥ 95th percentile; and for adolescents aged ≥13 years: ≥ 130/80 mmHg*) (23) and obesity based on sex-specific BMI-for-age (*≥ 95^th^ percentile*) (24). Other primary outcomes included elevated cardiovascular risk parameters: blood glucose > 100 mg/dL, LDL-C > 110 mg/dL, TG > 90 mg/dL, total cholesterol > 170 mg/dL, non-HDL-C > 120 mg/dL, but increasing HDL-C level > 40 mg/dL (25). In addition to the previous variables, the following continuous variables were used as secondary outcomes, including SBP (mmHg), DBP (mmHg), glucose (mg/dL), LDL-C (mg/dL), TG (mg/dL), total cholesterol (mg/dL), HDL-C (mg/dL), non-HDL-C (mg/dL), sex-specific BMI-for-age (z-score), and WHtR over the follow-up.

### 2.6 Statistical analyses

The characteristics of the study sample were described in numbers, means, percentages (%), and standard deviations (SD). The Kolmogorov–Smirnov test was used to check their normality. The Kruskal–Wallis and Pearson chi-squared tests were used to test quantitative and categorical variables. Orthogonal polynomial contrasts evaluated linear trends. Missing data on variables of interest ranged from <0.1% (high BP at baseline) to 3.4, 7.8, and 15.1% in total cholesterol, HDL-C (at baseline, 2 years, and 4 years of follow-up, respectively); while missing values for BMI z-score were 0.1, 7.6, and 14.5% (at baseline, 2 years, and 4 years of follow-up, respectively). We performed the last observation carried forward method to account for missing data (26). Cox regression analyses evaluated the association (hazard ratios - HRs) and 95% confidence intervals (CIs) between time and event (among 4 years of follow-up). The cumulative average PHDI score was calculated from each follow-up period, and the following outcomes were studied: risk of high BP, obesity, glucose >100 mg/dL, total cholesterol >170 mg/dL, HDL-C > 40 mg/dL, and non-HDL-C > 120 mg/dL. The clustering approach was considered across municipalities (Barcelona/Madrid) and schools. Outcome adjustments were conducted using two multivariable models. Multivariable model A was adjusted for gender (male/female), baseline age (11–12 years/13–14 years), parental education level (primary/secondary/academic-graduate), randomized group (control/long-term intervention/short-term intervention), and Tanner maturation stage (from I to V). Multivariable model B was adjusted for variables of model A, plus the following baseline variables: adolescent high BP status (yes/no), BMI-for-age (≥5th to <85th percentile/≥85^th^ to <95th percentile/≥95^th^ percentile), MVPA 60 min-day (yes/no), sleep duration (hours, continuous), and energy intake (kcal/day, continuous). For BP analysis, the model B further included dietary sodium and potassium ratio (continuous) and dietary calcium (mg/day, continuous), while HDL-C was further adjusted by dietary saturated fat (mg/day) using the energy-adjusted residual method (19). Likelihood ratio tests for interaction explored potential interactions between adherence to PHDI and gender. HRs were also estimated for outcomes for every 20-point increase in the PHDI. Furthermore, we also studied dose–response models using restricted cubic splines (RCS) Cox regression with 5 knots (27) to assess the relationship between the cumulated adherence to PHDI and the previously mentioned outcomes, adjusting for model B.

As a secondary analysis, we analyzed PHDI adherence and longitudinal changes in cardiometabolic parameters (BP, glucose, LDL-C, TG, total cholesterol, HDL-C, non-HDL-C, BMI z-score, and WHtR) by using multilevel linear mixed models during three visits over the 4 years of follow-up. Fitted models were clustered across recruitment municipalities and schools, and two-level random intercepts (municipality and participant). Models were adjusted (A and B) using the same covariates described in the Cox models but including the following time-varying variables: adolescent BMI-for-age, MVPA 60 min-day, sleep duration, energy intake, and saturated fat intake. *p*-values <0.05 were considered significant. Analyses were performed using Stata (Stata-Corp LP, TX, USA) version 16.1.

## 3 Results

Study population characteristics, lifestyle, food consumption, and nutrient intake according to the adherence to PHDI are described in Tables 1, 2. At baseline, approximately half of the participants were female (49.1%), aged 12.0 years, with a maternal migrant background of approximately 20%, and a mean BMI of 20.3 kg/m^2^. Out of a maximum of 140 points, adolescents had a mean score of 89.9 ± 13.1, with minimum and maximum scores of 54.5 points and 131.5 points, respectively (Supplementary Figure 1). When comparing the PHDI scores by gender, girls had a mean of 91.1 ± 13.5 points, while boys had a mean of 88.8 ± 12.7 points. No differences were found according to the place of residence between Barcelona and Madrid (89.2 ± 13.1 points and 91.2 ± 13.1 points, respectively).

**Table 1:** Baseline characteristics of the participants according to the Planetary Health Diet Index (PHDI) in the SI! Program.

Characteristics	Whole sample	Q1	Q2	Q3	Q4	*p*-value ^†^	*p*-trend
		< 80.5	80.5–90	90.1–98.5	> 98.5		
	*n* = 886	*n* = 223	*n* = 231	*n* = 213	*n* = 219		
**Demographics**							
Age (y)	12.0 (0.4)	12.0 (0.4)	12.0 (0.4)	12.0 (0.4)	12.0 (0.4)	0.88	0.88
Gender							
Male	451 (50.9%)	120 (54.0%)	120 (52.0%)	110 (51.6%)	101 (46.1%)	0.41	0.12
Female	435 (49.1%)	103 (46.2%)	111 (48.1%)	103 (48.4%)	118 (53.9%)		
Tanner stages^‡^							
I	13 (1.5%)	4 (1.8%)	3 (1.3%)	3 (1.4%)	3 (1.4%)	0.73	0.74
II	181 (20.5%)	43 (19.3%)	45 (19.6%)	42 (19.7%)	51 (23.5%)		
III	430 (48.7%)	112 (50.2%)	117 (50.9%)	101 (47.4%)	100 (46.1%)		
IV	220 (24.9%)	50 (22.4%)	58 (25.2%)	61 (28.6%)	51 (23.5%)		
V	39 (4.4%)	14 (6.3%)	7 (3.0%)	6 (2.8%)	12 (5.5%)		
Annual household income							
Low	240 (28.7%)	60 (28.4%)	57 (25.7%)	52 (26.0%)	71 (35.0%)	0.17	0.33
Average	251 (30.0%)	59 (28.0%)	78 (35.1%)	64 (32.0%)	50 (24.6%)		
High	345 (41.3%)	92 (43.6%)	87 (39.2%)	84 (42.0%)	82 (40.4%)		
Municipality							
Barcelona	596 (67.3%)	158 (70.9%)	159 (68.8%)	141 (66.2%)	138 (63.0%)	0.33	0.06
Madrid	290 (32.7%)	65 (29.1%)	72 (31.2%)	72 (33.8%)	81 (37.0%)		
Parental education							
Primary	39 (17.7%)	30 (13.2%)	32 (15.7%)	30 (14.2%)	35 (17.3%)	0.11	0.021*
Secondary	99 (45.0%)	85 (37.6%)	78 (38.2%)	73 (34.6%)	83 (41.1%)		
Academic/graduate	82 (37.3%)	111 (49.1%)	94 (46.1%)	108 (51.2%)	84 (41.6%)		
Maternal migrant background	163 (19.7%)	32 (15.2%)	34 (15.5%)	41 (20.8%)	56 (28.0%)	<0.01**	<0.001***
**Lifestyle and risk factors**							
MVPA ≥ 60 min/day	599 (67.6%)	154 (69.1%)	153 (66.2%)	148 (69.5%)	144 (65.8%)	0.78	0.64
Sleep time, hours	7.2 (1.0)	7.2 (0.9)	7.2 (0.9)	7.1 (1.1)	7.2 (1.0)	0.99	0.99
Body weight, kg	48.9 (11.0)	47.9 (11.7)	47.9 (10.2)	50.1 (10.4)	49.6 (11.6)	0.07	0.07
BMI, kg/m^2^	20.3 (3.7)	20.1 (4.0)	19.9 (3.4)	20.6 (3.7)	20.4 (3.8)	0.17	0.17
BMI, z-score	0.4 (0.4)	0.3 (1.0)	0.3 (0.9)	0.5 (1.0)	0.4 (1.0)	0.14	0.13
BMI status							
Healthy weight	617 (69.7%)	161 (72.5%)	171 (71.0%)	141 (66.2%)	144 (65.8%)	0.44	0.08
Overweight	162 (18.3%)	35 (15.8%)	38 (16.4%)	45 (21.1%)	44 (20.1%)		
Obesity	83 (9.4%)	20 (9.0%)	16 (6.9%)	24 (11.3%)	23 (10.5%)		
Waist circumference, cm	71.9 (10.1)	71.6 (11.0)	70.9 (9.1)	72.9 (10.1)	72.2 (10.4)	0.21	0.21
High BP status^§^	115 (13.0%)	33 (14.8%)	29 (12.7%)	28 (13.2%)	25 (11.4%)	0.77	0.34
SBP, mmHg	109.1 (10.5)	108.3 (11.1)	109.0 (10.3)	110.3 (10.1)	109.3 (10.7)	0.28	0.28
High BP adolescents^¶^	123.8 (9.0)	125.9 (9.0)	120.8 (9.0)	125.1 (9.2)	123.2 (8.0)	0.12	0.12
DBP, mmHg	65.7 (8.6)	65.6 (8.8)	65.8 (8.9)	65.7 (8.3)	65.8 (8.3)	0.99	0.99
High BP adolescents^¶^	77.7 (8.6)	77.1 (9.3)	77.7 (9.1)	76.0 (8.5)	80.1 (7.1)	0.36	0.36
Blood glucose level, mg/dL	103.4 (17.0)	102.3 (11.0)	105.7 (26.9)	102.1 (12.4)	103.3 (10.8)	0.10	0.10
HDL-C, mg/dL	62.9 (16.0)	64.1 (16.4)	63.9 (15.6)	60.8 (16.0)	62.6 (15.5)	0.13	0.13
LDL-C, mg/dL	78.3 (26.0)	79.8 (26.0)	77.4 (25.4)	78.8 (26.5)	77.4 (26.0)	0.80	0.80
Total cholesterol, mg/dL	152.9 (33.2)	154.8 (33.8)	153.2 (32.4)	150.5 (32.7)	153.2 (34.1)	0.61	0.61
Triglycerides, mg/dL	78.0 (40.0)	80.6 (42.5)	76.1 (43.1)	78.1 (37.8)	77.4 (35.9)	0.70	0.70
Non-HDL-C, mg/dL	90.1 (29.0)	90.9 (29.3)	89.3 (27.2)	89.6 (29.2)	90.7 (30.6)	0.93	0.93

Data are given as means (SDs) for continuous variables and *n* (%) for categorical variables.

*p*-value for comparisons across PHDI (quartiles).

*p*-value and *p*-trend < 0.05 considered significant, values shown in bold are statistically significant (*p ≤ 0.05; **p ≤ 0.01; ***p ≤ 0.001).

^†^Data normality was verified by the Kolmogorov–Smirnov test. *p*-value based on one-way ANOVA or Kruskal–Wallis test, while χ^2^ test was used for categorical variables.

^‡^Puberty development was evaluated using pictograms, with stage I defined as prepubertal and stage V defined as mature.

^§^High BP based on adolescents <13 years: ≥ 95th percentile; and for adolescents aged ≥13 years: ≥ 130/80 mmHg.

^¶^In the adolescent subgroup with high BP at baseline.

BP, Blood Pressure; BMI, Body Mass Index; DBP, Diastolic blood pressure; HDL-C, High-density lipoprotein cholesterol; LDL-C, Low-density lipoprotein cholesterol; MVPA, Moderate to vigorous physical activity; PHDI, Planetary Health Diet Index; SBP, Systolic blood pressure; SD, Standard deviation.

**Table 2:** Baseline dietary pattern of the participants according to the PHDI in the SI! Program.

Characteristics	Whole sample	Q1	Q2	Q3	Q4	*p*-value^†^	*p*-trend
		< 80.5	80.5–90	90.1–98.5	> 98.5		
	*n* = 886	*n* = 223	*n* = 231	*n* = 213	*n* = 219		
Nutritional intake							
Total energy intake, kcal/day	2,532 (597.6)	2,605 (605.1)	2,567 (590.2)	2,516 (607.8)	2,435 (577.4)	0.018*	0.02*
Carbohydrates, g/day	255.8 (72.0)	257.6 (73.4)	256.1 (71.4)	257.8 (71.8)	251.4 (71.6)	0.77	0.77
Proteins, g/day	120.8 (33.1)	128.7 (34.4)	125.4 (31.0)	118.9 (34.1)	109.6 (29.9)	<0.001***	<0.001***
Proteins, g/kg/day	2.6 (1.0)	2.8 (1.0)	2.8 (1.0)	2.5 (0.9)	2.3 (0.8)	<0.001***	<0.001***
Animal proteins, g/day	90.2 (29.8)	100.6 (31.3)	95.6 (27.0)	87.7 (30.2)	76.3 (24.8)	<0.001***	<0.001***
Plant proteins, g/day	30.6 (9.7)	28.0 (9.1)	29.8 (8.8)	31.2 (9.2)	33.2 (10.8)	<0.001***	<0.001***
Fat, g/day	23.4 (8.3)	23.4 (8.8)	22.2 (8.0)	24.1 (8.2)	23.9 (8.3)	0.08	0.08
Monounsaturated fats, g/day	48.5 (16.1)	48.3 (15.1)	49.5 (15.5)	47.9 (16.4)	48.1 (17.3)	0.73	0.73
Polyunsaturated fats, g/day	19.5 (6.6)	19.6 (6.3)	19.3 (6.6)	19.4 (7.0)	19.9 (6.7)	0.80	0.80
Saturated fat, g/day	68.0 (21.2)	40.3 (12.6)	37.6 (10.7)	35.5 (11.2)	33.0 (9.7)	<0.001***	<0.001***
Fiber, g/day	29.4 (10.7)	23.9 (8.8)	28.4 (9.1)	30.3 (9.0)	35.4 (12.2)	<0.001***	<0.001***
Sodium intake, mg/day	3,400 (1,066)	3,564 (1,171)	3,457 (994.7)	3,433 (1,055)	3,141 (992.9)	<0.001***	<0.001***
Potassium intake, mg/day	4,479 (1304)	4,142 (1226)	4,496 (1267)	4,758 (1,400)	4,758 (1,400)	<0.001***	<0.001***
Dietary sodium:potassium ratio	0.8 (0.3)	0.9 (0.3)	0.8 (0.3)	0.8 (0.2)	0.7 (0.2)	<0.001***	<0.001***
Calcium, mg/day	1,011 (388.2)	1,041 (422.5)	1,011 (375.5)	1,001 (387.4)	990.3 (366.2)	0.56	0.56
Iron, mg/day	18.0 (4.9)	17.4 (4.9)	18.2 (4.7)	18.1 (4.7)	18.5 (5.2)	0.08	0.08
Zinc, mg/day	13.7 (3.7)	14.1 (3.9)	14.0 (3.7)	13.6 (3.5)	13.1 (3.6)	**0.017**	**0.02**
Vitamin B12, mcg/day	9.5 (6.6)	10.2 (7.3)	10.0 (7.0)	9.0 (6.8)	8.6 (5.1)	**0.03**	**0.03**
Food intake (g/day)							
Dairy^‡^	403.9 (250.2)	472.2 (306.5)	413.4 (232.5)	385.3 (219.7)	342.5 (213.5)	<0.001***	<0.001***
Cheese	14.5 (15.2)	15.8 (15.4)	13.6 (13.7)	14.7 (15.5)	13.9 (16.2)	0.42	0.42
Meat	179.2 (91.8)	220.8 (95.9)	197.0 (80.5)	171.1 (87.0)	125.9 (75.0)	<0.001***	<0.001***
Beef	28.7 (23.9)	33.2 (26.6)	33.3 (23.9)	29.0 (23.6)	19.1 (18.2)	<0.001***	<0.001***
Pork	85.3 (48.6)	102.5 (53.8)	94.2 (47.5)	82.6 (44.1)	61.0 (37.2)	<0.001***	<0.001***
Poultry	66.9 (53.2)	83.4 (45.7)	72.1 (43.6)	63.0 (59.6)	48.6 (57.1)	<0.001***	<0.001***
Eggs	22.3 (12.5)	23.5 (15.4)	21.9 (9.5)	23.0 (11.1)	20.8 (12.8)	0.11	0.11
Seafoods	86.9 (54.2)	77.1 (52.4)	92.1 (56.5)	87.4 (46.1)	90.8 (59.6)	0.01**	0.01**
Potatoes	52.8 (38.3)	58.9 (41.1)	52.8 (38.0)	55.0 (40.2)	44.2 (32.0)	<0.001***	<0.001***
Legumes	60.9 (44.4)	49.7 (49.1)	57.9 (40.6)	63.8 (35.1)	72.6 (48.5)	<0.001***	<0.001***
Nuts	11.1 (13.50)	6.3 (8.2)	9.7 (11.6)	11.5 (12.6)	17.1 (17.6)	<0.001***	<0.001***
Vegetables	208.2 (152.0)	128.2 (99.3)	190.8 (126.1)	220.7 (149.6)	295.7 (174.7)	<0.001***	<0.001***
Dark & green vegetables	124.3 (91.7)	78.3 (61.9)	117.3 (77.5)	131.1 (89.7)	172.1 (107.4)	<0.001***	<0.001***
Red & orange vegetables	65.5 (67.3)	42.3 (44.8)	57.9 (57.9)	69.9 (67.2)	92.8 (83.9)	<0.001***	<0.001***
Fruits	334.4 (247.3)	237.0 (218.1)	333.9 (242.8)	354.3 (199.6)	414.8 (286.9)	<0.001***	<0.001***
Refined cereals	113.2 (69.5)	129.3 (74.4)	117.2 (69.3)	115.0 (71.4)	90.6 (56.1)	<0.001***	<0.001***
Whole grains	18.3 (31.8)	4.5 (12.6)	12.9 (24.0)	20.8 (34.4)	35.4 (40.9)	<0.001***	<0.001***
Added sugars	25.6 (16.5)	29.0 (19.4)	27.8 (17.7)	24.3 (14.4)	20.9 (12.2)	<0.001***	<0.001***
Total olive oil	16.8 (14.7)	13.5 (11.8)	17.0 (14.6)	17.4 (13.3)	19.4 (17.8)	<0.001***	<0.001***
Extra-virgin olive oil	9.24 (9.8)	7.46 (8.2)	8.8 (9.7)	9.5 (9.7)	11.3 (11.2)	<0.001***	<0.001***
Sunflower oil	1.4 (2.9)	1.1 (2.4)	1.3 (2.9)	1.3 (2.9)	1.7 (3.4)	0.19	0.19
Butter	1.0 (1.9)	0.77 (1.8)	1.0 (1.8)	1.3 (2.0)	0.95 (1.81)	**0.04**	**0.04**
Margarine	0.8 (1.8)	0.8 (1.7)	0.8 (1.9)	0.9 (1.9)	0.7 (1.5)	0.75	0.75
Water	876.7 (428.6)	847.3 (442.9)	885.7 (435.8)	853.4 (419.0)	919.9 (414.3)	0.26	0.26
Fruit juices	54.6 (78.0)	42.3 (65.7)	59.0 (92.2)	59.9 (79.4)	57.4 (70.6)	0.06	0.06
Plant milk^¶^	10.3 (48.3)	2.7 (19.9)	3.1 (19.4)	13.6 (58.5)	22.6 (71.4)	<0.001***	<0.001***
Sugar-sweetened beverages	33.7 (71.8)	41.7 (93.1)	30.5 (57.9)	39.9 (81.1)	22.9 (43.6)	0.02*	0.02*

Data are given as means (SDs).

*p*-value for comparisons across PHDI (quartiles).

*p*-value and *p*-trend < 0.05 considered significant, values shown in bold are statistically significant (*p ≤ 0.05; **p ≤ 0.01; ***p ≤ 0.001).

^†^Data normality was verified by the Kolmogorov–Smirnov test. *p*-value based on one-way ANOVA or Kruskal–Wallis test.

^‡^Including milk (liquid and powder), condensed milk, and yogurt.

^¶^Including soy, almond, or rice milks.

PHDI, Planetary Health Diet Index; SD, Standard deviation.

Regarding the PHDI scores, low adherence was identified for key items such as soy and soy foods (90% below 5 points), red and processed meat (74.3% below 5 points), pulses (90% below 5 points), whole grains (40.9% below 5 points), unsaturated oils (40.9% below 5 points), and peanuts and tree nuts (40.1% below 5 points). The baseline proportion of these items within the total PHDI score is shown in Supplementary Figure 2. Reasonably, there were significant increases and decreases in most reported dietary components and food items across the PHDI quartiles (Table 2; Supplementary Figure 3). When examining PHDI attitudes toward adherence in families with migrant mothers, there was little to no difference compared to other families (Supplementary Figure 4). Participants in the highest quartile consumed more dietary fiber, potassium, and seafoods, and consumed almost double amounts of legumes, nuts, vegetables (dark & green/red & orange), and fruits compared to the lowest quartile. Increased consumption of whole grains, olive oil, and plant milks was observed in the highest quartile, while dairy, animal proteins (beef, pork, and poultry), potatoes, added sugars, and sugar-sweetened beverages were less consumed compared to the lowest quartile of the PHDI. As dietary patterns shifted toward higher PHDI adherence, participants consumed fewer calories from proteins (plant-based and animal-based proteins), along with reduced sodium and increased potassium intake. Differences in calcium, zinc, iron, and vitamin B12 intake were identified, with levels becoming more pronounced by gender, according to the PHDI (Supplementary Figure 5).

**Figure 2:**
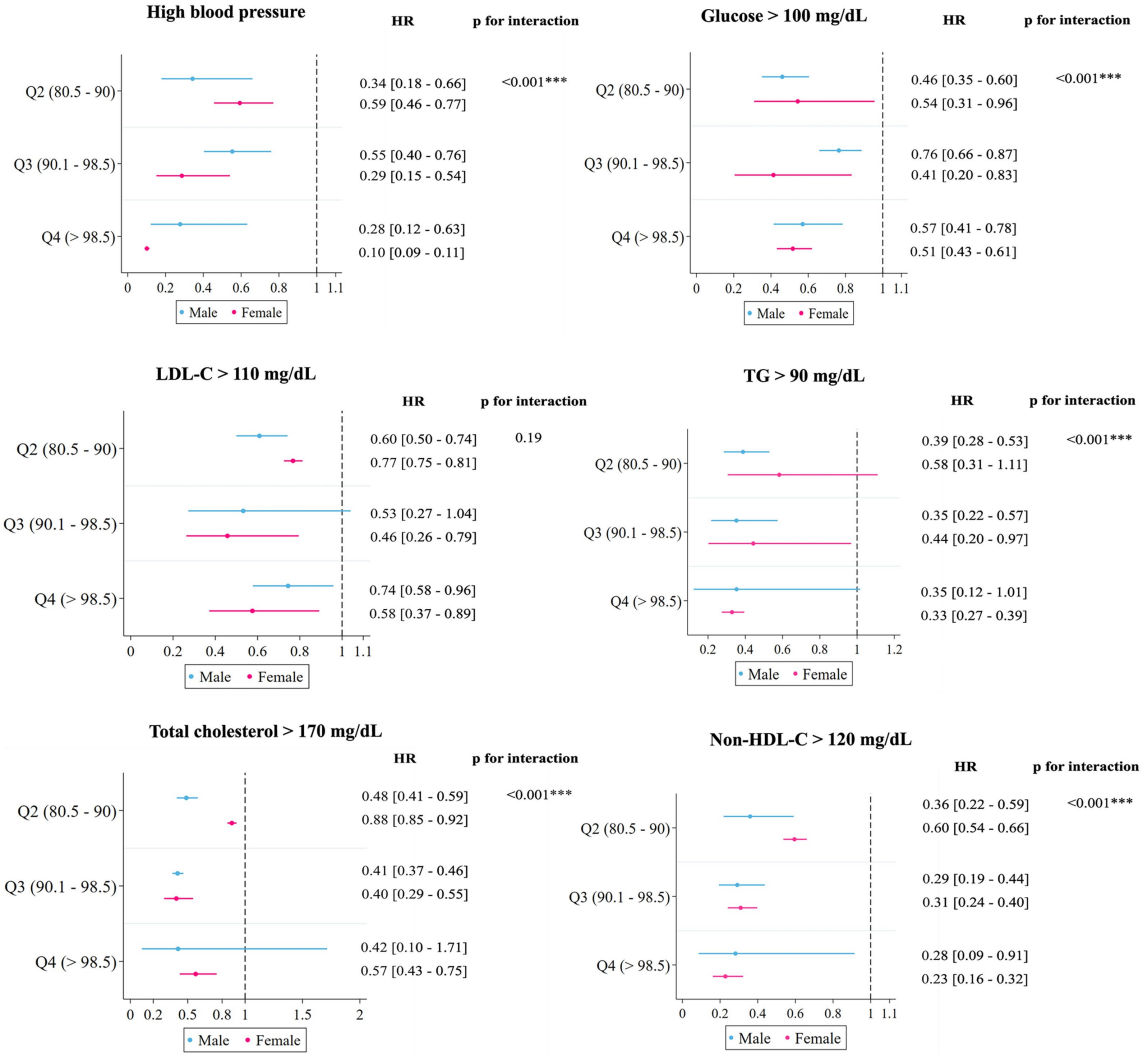
Planetary Health Dietary Index and hazard ratios (HRs) with 95% CI in 886 participants in the SI! Program, based on the fully adjusted model by gender. HDLC, High-density lipoprotein cholesterol; HR, Hazard ratio; LDL-C, Low-density lipoprotein cholesterol; TG, Triglycerides.

The mean follow-up of participants was 3.5 years. In the fully adjusted Cox regression models (Table 3), significant linear trends were observed, indicating a reduced risk of high BP, elevated plasma glucose, TG, total cholesterol, and non-HDL-C with increased PHDI adherence. A significant inverse association with high adherence to PHDI, whether evaluated in quartiles, and for each 20-point increase (Table 3) was observed for high BP, and increased glucose, TG, total cholesterol, and non-HDL-C. In contrast, no significant reductions for LDL-C, obesity (HR: 0.73 [95% CI: 0.48, 1.11]; *p*-value = 0.14), and HDL-C (HR: 1.86 [95% CI: 0.42, 8.27]; *p-*value = 0.42) were found by 20-point increase. When comparing the higher vs. lower PHDI adherence _(Q4 vs Q1)_, the risk of high BP was significantly reduced by 81% (HR: 0.19 [95% CI: 0.11, 0.34]), plasma glucose by 47% (HR: 0.53 [95% CI: 0.48, 0.58]), TG by 66% (HR: 0.34 [95% CI: 0.18, 0.65]), total cholesterol by 51% (HR: 0.49 [95% CI: 0.34, 0.69]), and non-HDL-C by 74% (HR: 0.26 [95% CI: 0.13, 0.50]). Comparisons between the standardized diet and the reported diet showed no variations in the estimators (Supplementary Table 1). Limitations to study obesity and HDL-C > 40 mg/dL were attributable to the low incidence, 27 cases of obesity that made up approximately 3.3%; while out of the 35 participants who had HDL-C below 40 at baseline, 20 experienced an increase in their HDL-C concentrations. PHDI_(Q4 vs Q1)_ gender stratified analyses for elevated cardiometabolic parameters achieved statistical significance in both genders, mainly in girls (Figure 2). The results of the RCS Cox regression showed a non-significant J-shaped association (*p*-value for non-linearity) between the PHDI and outcomes of interest (Supplementary Figure 6).

**Table 3:** Cox regression models^†^ for the cumulative average PHDI, risk of new-onset high blood pressure, and elevated cardiometabolic risk biomarkers in the SI! Program.

	Q1	Q2				Q3				Q4					Hazard ratio of PHDI for 20-point increase^‡^		
	< 80.5	80.5–90				90.1–98.5				> 98.5							
High blood pressure																	
Cases/Person-years (219/2890)	101/652	48/720				46/772				24/746							
Incidence rate	0.15	0.07				0.06				0.03							
		**HR**	**CI (95%)**		***p*-value**	**HR**	**CI (95%)**		***p*-value**	**HR**	**CI (95%)**		***p*-value**	***p*-trend**	**HR**	**CI (95%)**	***p*-value**
Model A	Ref.	0.45	0.43	0.47	<0.001***	0.41	0.27	0.64	<0.001***	0.21	0.10	0.45	<0.001***	<0.001***			
Model B	Ref.	0.48	0.43	0.54	<0.001***	0.43	0.29	0.65	<0.001***	0.19	0.11	0.34	<0.001***	<0.001***	0.43	0.33 0.56	<0.001***
Glucose > 100 mg/dL																	
Cases/Person-years (169/1020)	60/220	41/314				38/256				30/230							
Incidence rate	0.27	0.13				0.15				0.13							
		**HR**	**CI (95%)**		***p*-value**	**HR**	**CI (95%)**		***p*-value**	**HR**	**CI (95%)**		***p*-value**	***p*-trend**	**HR**	**CI (95%)**	***p*-value**
Model A	Ref.	0.52	0.38	0.72	<0.001***	0.57	0.56	0.57	<0.001***	0.53	0.53	0.53	<0.001***	<0.001***			
Model B	Ref.	0.52	0.34	0.78	<0.01**	0.56	0.50	0.62	<0.001***	0.53	0.48	0.58	<0.001***	<0.01**	0.74	0.69 0.80	<0.001***
LDL-C ≥ 110 mg/dL																	
Cases/Person-years (314/1964)	100/448	79/498				61/526				74/492							
Incidence rate	0.22	0.16				0.12				0.15							
		**HR**	**CI (95%)**		***p*-value**	**HR**	**CI (95%)**		***p*-value**	**HR**	**CI (95%)**		***p*-value**	***p*-trend**	**HR**	**CI (95%)**	***p*-value**
Model A	Ref.	0.74	0.72	0.75	<0.001***	0.52	0.23	1.17	0.11	0.72	0.45	1.15	0.17	0.11			
Model B	Ref.	0.71	0.61	0.81	<0.001***	0.49	0.25	0.98	0.04*	0.68	0.46	1.01	0.06	0.04*	0.78	0.54 1.14	0.20
TG > 90 mg/dL																	
Cases/Person-years (242/2802)	105/664	54/700				44/734				39/704							
Incidence rate	0.16	0.07				0.06				0.06							
		**HR**	**CI (95%)**		***p*-value**	**HR**	**CI (95%)**		***p*-value**	**HR**	**CI (95%)**		***p*-value**	***p*-trend**	**HR**	**CI (95%)**	***p*-value**
Model A	Ref.	0.50	0.42	0.56	<0.001***	0.38	0.28	0.50	<0.001***	0.35	0.19	0.63	<0.001***	<0.001***			
Model B	Ref.	0.50	0.42	0.59	<0.001***	0.39	0.31	0.48	<0.001***	0.34	0.18	0.65	<0.001***	<0.01**	0.53	0.41 0.68	<0.001***
Total cholesterol > 170 mg/dL																	
Cases/Person-years (211/2136)	83/532	51/498				36/586				41/520							
Incidence rate	0.16	0.10				0.06				0.08							
		**HR**	**CI (95%)**		***p*-value**	**HR**	**CI (95%)**		***p*-value**	**HR**	**CI (95%)**		***p*-value**	***p*-trend**	**HR**	**CI (95%)**	***p*-value**
Model A	Ref.	0.68	0.57	0.80	<0.001***	0.38	0.31	0.47	<0.001***	0.50	0.26	0.94	0.031*	0.031*			
Model B	Ref.	0.66	0.57	0.76	<0.001***	0.39	0.39	0.39	<0.001***	0.49	0.34	0.69	<0.001***	<0.001***	0.62	0.53 0.74	<0.001***
Non-HDL-C ≥ 120 mg/dL																	
Cases/Person-years (202/2636)	95/632	46/658				33/690				28/656				95/632			
Incidence rate	0.15	0.07				0.05				0.04				0.15			
		**HR**	**CI (95%)**		***p*-value**	**HR**	**CI (95%)**		***p*-value**	**HR**	**CI (95%)**		***p*-value**	***p*-trend**	**HR**	**CI (95%)**	***p*-value**
Model A	Ref.	0.48	0.45	0.51	<0.001***	0.36	0.28	0.47	<0.001***	0.22	0.10	0.53	<0.001***	<0.001***			
Model B	Ref.	0.49	0.47	0.53	<0.001***	0.32	0.28	0.36	<0.001***	0.26	0.13	0.50	<0.001***	<0.001***	0.45	0.31 0.66	<0.001***

Cox regression (clustering at the recruitment municipality level and school) was used to conduct this analysis. Multivariable model A: gender (male/female), age (11–12 years/13–14 years), parental education (primary/secondary/academic-graduate), randomized group (control/long-term intervention/short-term intervention), baseline Tanner maturation stage (from I to V). Multivariable model B: variables of model A plus, adolescent high blood pressure status (yes/no), adolescent BMI-for-age (≥5th to <85th percentile/≥85th to <95th percentile/≥95th percentile), moderate to vigorous physical activity 60 min-day (yes / no), sleep duration (hours, continuous), energy intake (kcal/day, continuous). For high BP analysis model B, further adjustment included: dietary sodium and potassium ratio (continuous), and calcium (mg/day, continuous); both adjusted for total energy using the residual method.

*p*-value <0.05 considered significant, values shown in bold are statistically significant (*p ≤ 0.05; **p ≤ 0.01; ***p ≤ 0.001).

^†^ Number of participants not having the condition or the elevated cardiovascular marker at baseline: High BP: *n* = 769; Glucose: *n* = 320; LDL-C: 597; TG: *n* = 509; Total cholesterol: *n* = 856; Non-HDL-C: *n* = 719.

^‡^ Fitted according to model B.

CI, Confidence interval; HR, Hazard ratio; LDL-C, Low-density lipoprotein cholesterol; PHDI, Planetary Health Diet Index; TG, Triglycerides.

Results of the linear mixed models assessing changes in cardiometabolic risk biomarkers and the PHDI are shown in Table 4. The PHDI _(Q4 vs Q1)_ was inversely associated with reductions of glucose (−5.23 mg/dL [95% CI: −10.35, −0.10]), TG (−2.48 mg/dL [95% CI: −3.65, −1.30]), and BMI z-score (−0.02 [95% CI: −0.03, 0.00]). In addition, we found statistically significant inverse associations between PHDI _(Q3 vs Q1)_, SBP (−3.83 mmHg [95% CI: −3.98, −3.67]), and DBP (−3.83 mmHg [95% CI: −3.98, −3.67]) in participants with high BP at baseline. Overall, trends were observed in the other cardiometabolic risk parameters; however, no association was observed when comparing them as continuous variables.

**Table 4:** Associations between changes of cardiometabolic risk biomarkers^†^ and the PHDI in the SI! Program during 4 years of follow-up.

	Q1	Q2				Q3				Q4				*p*-trend	ICC
	< 80.5	80.5–90				90.1–98.5				> 98.5					
		β	CI (95%)		*p*-value	β	CI (95%)		*p*-value	β	CI (95%)		*p*-value		
SBP (mgHg)															
Model A	Ref.	0.83	−0.02	1.68	0.054	1.00	−0.05	2.06	0.06	1.30	0.02	2.59	0.05	0.11	0.45
Model B	Ref.	1.79	−1.81	5.39	0.33	1.01	1.91	2.12	<0.001***	1.41	−0.61	3.44	0.17	0.32	0.35
DBP (mgHg)															
Model A	Ref.	0.20	−0.54	0.95	0.59	−0.32	−0.97	0.33	0.34	0.22	−0.70	1.14	0.63	0.52	0.45
Model B	Ref.	0.71	0.41	1.03	<0.001***	0.36	−1.34	2.07	0.68	0.19	−2.43	2.80	0.89	0.89	0.35
SBP (mgHg) ^‡^															
Model A	Ref.	−4.24	−5.52	−2.96	<0.001***	0.23	−1.33	1.78	0.78	−0.84	−2.33	0.66	0.27	0.78	0.43
Model B	Ref.	−3.66	−6.05	−1.28	<0.01**	−3.83	−3.98	−3.67	<0.001***	−3.74	−9.91	2.43	0.24	0.23	0.13
DBP (mgHg) ^†^															
Model A	Ref.	−1.51	−3.76	0.73	0.19	−2.21	−2.63	−1.79	<0.001***	−1.41	−6.50	3.69	0.59	0.59	0.21
Model B	Ref.	−3.66	−6.05	−1.28	<0.01**	−3.83	−3.98	−3.67	<0.001***	−3.74	−9.91	2.43	0.24	0.23	0.13
Glucose (mg/dL)															
Model A	Ref.	−0.96	−3.77	1.84	0.50	−1.69	−3.67	0.28	0.09	−1.68	−4.34	0.98	0.22	0.50	0.25
Model B	Ref.	−4.18	−9.79	1.44	0.15	−4.91	−9.66	−0.15	0.043*	−5.23	−10.35	−0.10	0.046*	0.15	0.20
LDL-C (mg/dL)															
Model A	Ref.	−0.52	−4.41	3.37	0.79	1.83	−1.96	5.63	0.34	−0.41	−2.69	1.87	0.72	0.79	0.59
Model B	Ref.	−0.72	−7.77	6.33	0.84	0.86	−5.45	7.16	0.79	−0.73	−6.58	5.12	0.81	0.84	0.55
TG (mg/dL)															
Model A	Ref.	1.56	0.62	2.49	<0.001***	−0.74	−5.80	4.32	0.77	0.89	−0.80	2.57	0.30	0.77	0.23
Model B	Ref.	0.84	−1.40	3.07	0.46	−2.06	−7.51	3.40	0.46	−2.48	−3.65	−1.30	<0.001***	0.46	0.17
Total cholesterol (mg/dL)															
Model A	Ref.	−2.04	−7.18	3.10	0.44	−0.51	−4.29	3.27	0.79	−1.42	−2.88	0.05	0.06	0.44	0.58
Model B	Ref.	−2.54	−11.11	6.03	0.56	−2.07	−8.62	4.48	0.54	−1.80	−6.53	2.93	0.46	0.56	0.55
HDL-C (mg/dL)															
Model A	Ref.	−1.54	−3.47	0.38	0.12	−1.80	−2.63	−0.97	<0.001***	−1.54	−2.89	−0.18	0.026*	0.12	0.61
Model B	Ref.	−1.66	−2.53	−0.78	<0.001***	−1.06	−5.44	3.32	0.64	−0.29	−0.71	0.12	0.16	0.64	0.57
Non-HDL-C (mg/dL)															
Model A	Ref.	−0.37	−4.50	3.75	0.86	1.44	−2.67	5.53	0.49	0.22	−4.46	4.91	0.93	0.92	0.57
Model B	Ref.	0.41	−0.92	1.74	0.55	−0.24	−0.79	0.30	0.37	0.23	−1.70	2.16	0.82	0.81	0.35
BMI (z-score)															
Model A	Ref.	0.00	−0.06	0.06	0.93	0.00	−0.07	0.07	0.99	0.00	−0.01	0.01	0.81	0.99	0.87
Model B	Ref.	−0.02	−0.08	0.03	0.36	−0.02	−0.06	0.03	0.41	−0.02	−0.03	0.00	0.04*	0.35	0.85
WHtR															
Model A	Ref.	−0.001	−0.004	0.001	0.31	−0.003	−0.004	−0.002	<0.001***	0.001	−0.004	0.005	0.81	0.81	0.81
Model B	Ref.	−0.003	−0.004	−0.001	<0.001***	−0.003	−0.007	0.001	0.18	−0.004	−0.007	0.001	0.06	0.18	0.55

Multilevel linear mixed models (clustering at recruitment municipality level and school) with municipality and participant considered as random intercepts. Multivariable model A: gender (male/female), age (11–12 years/13–14 years), parental education (primary/secondary/academic-graduate), maternal migrant background (yes /no), randomized group (control/long-term intervention/short-term intervention), baseline Tanner maturation stage (from I to V). Multivariable model B: variables of model A plus adolescent high blood pressure (yes/no), adolescent BMI-for-age (≥5th to <85th percentile/≥85th to <95th percentile/≥95th percentile), moderate to vigorous physical activity 60 min-day (yes/no), sleep duration (hours, continuous), energy intake (kcal/day, continuous). For the HDL-C analysis, dietary saturated fat (mg/day) was included, adjusted for total energy intake using the residual method.

*p*-value <0.05 considered significant, values shown in bold are statistically significant (*p ≤ 0.05; **p ≤ 0.01; ***p ≤ 0.001).

^†^ Number of participants within each quartile of the PHDI and cardiometabolic parameters over three time points (0, 2 and 4 years): SBP: *n* = 665, 667, 667 and 657; DBP: *n* = 665, 667, 667 and 657; and in those with high BP: SBP: *n* = 103, 81,78 and 87; DBP: *n* = 103, 81,78 and 87; Glucose: *n* = 649, 670, 657 and 641; LDL-C: *n* = 542, 584, 581 and 585; TG: *n* = 649, 670, 657 and 641; Total cholesterol: *n* = 648, 670, 657 and 641; HDL-C: *n* = 649, 670, 657 and 641; Non-HDL-C: *n* = 646, 669, 657 and 640; BMI (z-score): *n* = 668, 675, 664 and 650; WHtR: *n* = 669, 675, 664 and 650.

^‡^In those adolescents who have high BP at baseline, *n* = 115.

β, Beta regression coefficient; BMI, Body Mass Index; BP, Blood pressure; CI, Confidence interval; DBP, Diastolic Blood Pressure; HDL-C, High-density lipoprotein cholesterol; ICC, Intraclass correlation; LDL-C, Low-density lipoprotein cholesterol; PHDI, Planetary Health Diet Index; SBP, Systolic Blood Pressure; TG, Triglycerides; WHtR, Waist-to-height ratio.

## 4 Discussion

Our results indicate that a high PHDI adherence_(Q4 vs Q1)_ is associated with a risk reduction of high BP, increased plasma glucose, TG, total cholesterol, and non-HDL-C by 81, 47, 66, 51, and 74% respectively; and that high adherence to this dietary pattern is longitudinally associated with reductions of glucose (−5.23 mg/dL), TG (−2.48 mg/dL), and BMI z-score (−0.02) in adolescents. In contrast, when evaluating the PHDI _(Q4 vs Q1)_ and its relationship with LDL-C, total cholesterol, WHtR, SBP, and DBP as continuous variables in linear mixed models, no significant association is found. However, trends suggest a potential inverse association, which may reflect a healthier nutritional status. The analyses are robust, with most associations remaining significant after adjustments for various confounders and after sensitivity analyses. We found that when estimating the nutritional requirements (28) for energy, protein, zinc, iron, and vitamin B12 among adolescents with higher adherence to the PHDI, their intake was well within the recommended range. However, calcium intake was insufficient in female adolescents (4.4% below the requirements) among the highest quartiles. Promotion of calcium-rich plant foods and intake of mineral water as part of a healthy, balanced diet may facilitate reaching an adequate concentration of calcium.

Evidence that adherence to the PHDI is associated with changes in lipid profile, glucose, BP, or anthropometric parameters among adolescents is limited. In fact, consistent with our findings, a high PHDI adherence _(10-point increase)_ was associated with lower odds of hypertension (OR: 0.87 [95% CI: 0.79, 0.96]) (29), lower odds of increased total cholesterol (OR: 0.88 [95% CI: 0.78, 0.99]), and a higher Ideal Cardiovascular Health score. One study also found reductions in anthropometric parameters with PHDI adherence (30). Adjusted mean estimates revealed inverse associations between PHDI adherence and body weight (0.98 kg [95% CI: 0.97, 0.99]), BMI (0.99 kg/m^2^ [95% CI: 0.97, 0.99]), fat-free mass index (0.99 [95% CI: 0.99, 0.99]), waist circumference (0.99 cm [95% CI: 0.98, 0.99]), and body fat (0.98% [95% CI: 0.96, 0.99]) in an European cohort (30). Our results, regarding the excessive consumption of red and processed meats (Supplementary Figure 3) and adherence to the PHDI, are consistent with findings from studies in other young populations (31, 32). A surprising finding was that attitudes toward PHDI adherence were similar, including in families with a migrant background, as shown in Supplementary Figure 4. Some factors, such as socioeconomic level, duration of residence, and the role of country of origin (Mediterranean or non-Mediterranean), may promote dietary acculturation by adopting a healthy or a detrimental “Westernized” dietary pattern (33, 34).

The studies summarized below examine the effects of plant-based diets, such as the Mediterranean diet and the Dietary Approaches to Stop Hypertension (DASH) diet, on cardiometabolic biomarkers in pediatric populations, which underscore the relevance of this topic (35–40). These diets include food items similarly recommended in the Planetary Health Diet, but differing in the emphasis on sustainable eating. Results of a systematic review and meta-analysis evaluating the mean differences following the Mediterranean diet showed a significant inverse effect on SBP (−4.75 mm Hg [95% CI: −8.97,−0.52]), TG (−16.42 mg/dL [95% CI: −27.57, −5.27]), total cholesterol (−9.06 mg/dL [95% CI: −15.65, −2.48]), and LDL-C (−10.48 mg/dL [95% CI: −17.77, −3.19]), while increasing HDL-C (2.24 mg/dL [95% CI: 0.34, 4.14]) (41). Plant-rich diets have also shown to significantly reduce the odds of developing hypertension (OR, 0.63 [95% CI: 0.41, 0.97]) (35), mitigating the harmful effects of oxidative stress and inflammation, which are exacerbated by prolonged high BP (36). Significant reductions in SBP have been reported in two interventional studies: a decrease of 2.7 mmHg (*p*-value = 0.03) (36) and 10.4 mmHg (*p*-value <0.01) (37), both of them following the DASH diet. The Framingham Children’s study similarly observed reductions in mean SBP and DBP (4.60 mmHg and 1.12 mmHg, respectively) with a diet rich in fruits and vegetables (>4 servings/day) and low in dairy products (< 2 servings/day) (38). Other plant-based diets, such as the Mediterranean diet, have also shown significant improvements in lipid profile after a lifestyle intervention (39, 40). For example, reductions in total cholesterol (23.5 mg/dL), LDL-C (21.5 mg/dL), and non-HDL-C (21.5 mg/dL) were observed in children diagnosed with primary hypercholesterolemia (39), and in total cholesterol (25.5 mg/dL), LDL-C (22.0 mg/dL), and TG (12.0 mg/dL) (40).

Several reasons could explain the beneficial results in cardiometabolic biomarkers following this healthy pattern. One is the influence of low added sugars consumption (≤25 g/day of added sugars) (42), along with high consumption of whole grains, legumes, fruits, and vegetables, which has been shown to mitigate postprandial glucose excursions (41). A second reason underlies the consumption of phytonutrients, which have shown to activate *β*-oxidation, regulate satiety, and modulate energy intake (5, 43). These compounds can also induce thermogenesis in brown adipose tissue, and mobilize stored fat (5), while exhibiting antioxidant, anti-inflammatory, and immune-modulating properties (43, 44). A third reason involves the consumption of dietary fibers, such as β-glucans, arabinoxylans, and lignins, that play a crucial role in cardiometabolic health. They reduce the absorption of lipids and carbohydrates (45), control circulating LDL-C by inhibiting bile acid reabsorption (45), increase bacterial diversity (44), enhance intestinal barrier integrity (45), and promote the growth and metabolism of beneficial commensal Clostridia (Firmicutes) (46). High fermentation by major butyrate-producing bacteria, such as *Faecalibacterium*, further influences lipid metabolism through G-protein coupled receptors 41 and 43 (45, 47).

The strengths of the present study include its large-scale and long-term assessment of the PHDI and subsequent cardiometabolic risk screening during adolescence. Our results also addressed the controversies regarding nutrient intake, of particular concern in plant-based diets, in this population. Handling missing values in studied variables helped retain precise information for analysis, even after data loss occurred throughout the follow-up. Capillary blood measurements enabled us to efficiently assess cardiometabolic blood biomarkers, while wearable accelerometers offered objective measurements of physical activity and sleep. Repeated dietary assessments, along with the use of time-varying covariates to capture changes in dietary habits and health status, helped to reduce intraindividual variation over time. Furthermore, the validated FFQ used in this study showed good reproducibility and validity, and participants with extreme energy intakes were excluded to optimize the reliability of dietary information. By employing various data-driven analysis techniques, we were able to identify meaningful patterns and relationships.

However, the study also has some limitations. First, although BP readings were taken during the visits following standardized protocols, hypertension diagnoses were not confirmed through clinical records. Second, the use of proxy reporters for diet and other covariates may induce potential residual confounding in the analysis. The results may not be generalizable to other populations because participants were from a Mediterranean country. Caution should be exercised when interpreting glucose results, as some adolescents may have been assessed in a non-fasting state despite reporting that they were fasting. Additionally, the study design allows for the observation of the associations between PHDI adherence and outcomes, but it does not establish causality. It reflects the real-world conditions of the population rather than controlled experimental scenarios. Finally, the association between PHDI adherence, cardiometabolic health, and high BP was not a predefined endpoint of the SI! Program. This study makes the findings exploratory, requiring further research regarding PHDI, adolescent health, and educational strategies.

## 5 Conclusion

We observed strong inverse associations between higher PHDI adherence and the incidence of high BP, as well as several cardiometabolic risk factors. These findings suggest that a healthy plant-based diet, rich in phytochemicals and dietary fiber, promotes cardiometabolic health from an early age. This approach should mitigate the progression of cardiovascular diseases in younger populations while also reducing the environmental impact. Practical implications of these results include incorporating PHDI recommendations into school meals and education campaigns targeting adolescents and their families to promote the dual benefits of this diet for health and planetary sustainability.

## Data Availability

Data availability to external researchers is restricted to related project proposals upon request to the corresponding authors. Based on these premises, de-identified participant data will be available with publication after approval of the proposal by the steering committee and a signed data sharing agreement.
