# Supplementary material for: Is it time to align adolescent diets with the Planetary Health Diet? An observational study on early cardiovascular health
Source: Front Nutr. 2026 Feb 3;12:1739577. doi: 10.3389/fnut.2025.1739577 (PMC12911428; doi:10.3389/fnut.2025.1739577)
Supplement: Supplementary file 1 [file Table_1.docx]

Supplementary Material

**Is it Time to Align Adolescent Diets with the Planetary Health Diet?**

**An Observational Study on Early Cardiovascular Health.**

David Murcia-Lesmes^1,2^, Emily P. Laveriano-Santos^3,4^, Ramón Estruch^2,4,5^, Marina Corrado^1,2^, Camila Arancibia-Riveros^1,2^, Ana María Ruiz-León^2,4,5^, Rosa Casas^2,4,5^, Miguel Camafort^4,5^, Jesús Martínez-Gómez^6^, Amaya de Cos-Gandoy^6,7^, Patricia Bodega^6,7^, Gloria Santos-Beneit^6,7^, Juan M. Fernández-Alvira^6^, Rodrigo Fernández-Jiménez^6,8,9^, Rosa M. Lamuela-Raventós^1,2,4*^, Sara Castro-Barquero^2,4,5*^

^1^ Polyphenol Research Group. Departament de Nutrició, Ciències de l’Alimentació i Gastronomía, Facultat de Farmàcia i Ciències de l’Alimentació, Barcelona, Spain

^2^ Institut de Nutrició i Seguretat Alimentària (INSA-UB), Universitat de Barcelona, Barcelona, Spain

^3^ Barcelona Institute for Global Health (ISGlobal), Barcelona, Spain

^4^ Centro de Investigación Biomédica en Red de Fisiopatología de la Obesidad y Nutrición (CIBEROBN), Instituto de Salud Carlos III, Madrid, Spain

^5^ Department of Internal Medicine, Institut d’Investigacions Biomèdiques August Pi Sunyer (IDIBAPS), Hospital Clinic, University of Barcelona, Barcelona, Spain

^6^ Centro Nacional de Investigaciones Cardiovasculares (CNIC), Madrid, Spain

^7^ Foundation for Science, Health and Education (SHE Foundation), Barcelona, Spain

^8^ CIBER de Enfermedades Cardiovasculares (CIBERCV), Madrid, Spain

^9^ Hospital Universitario Clínico San Carlos, IdISSC, Madrid, Spain

***Correspondence:**

Rosa M. Lamuela-Raventós. Departament de Nutrició, Ciències de l’Alimentació i Gastronomía, Facultat de Farmàcia i Ciències de l’Alimentació. Institut de Nutrició i Seguretat Alimentària (INSA-UB), Universitat de Barcelona, Barcelona, Spain.

E-mail: [lamuela@ub.edu](mailto:lamuela@ub.edu)

***&***

Sara Castro-Barquero. Department of Internal Medicine, Institut d’Investigacions Biomèdiques August Pi Sunyer (IDIBAPS), Hospital Clinic, University of Barcelona, Barcelona, Spain.

E-mail: [sara.castro@ub.edu](mailto:sara.castro@ub.edu)

**Supplementary Figure 1.** Mean trends of PHDI with 95% CI during follow-up in the SI! Program. (possible range 0-140)

**
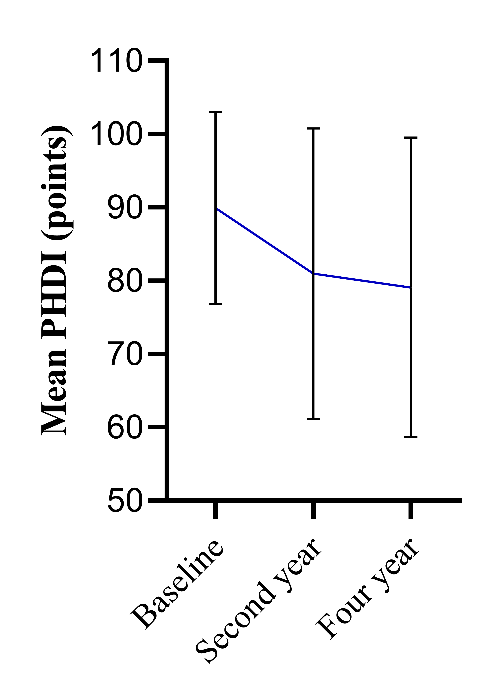
**

**Supplementary Figure 2.** Baseline proportion (%) of items within the total Planetary Health Diet Index (PHDI) score in the SI! Program, *n* = 886.


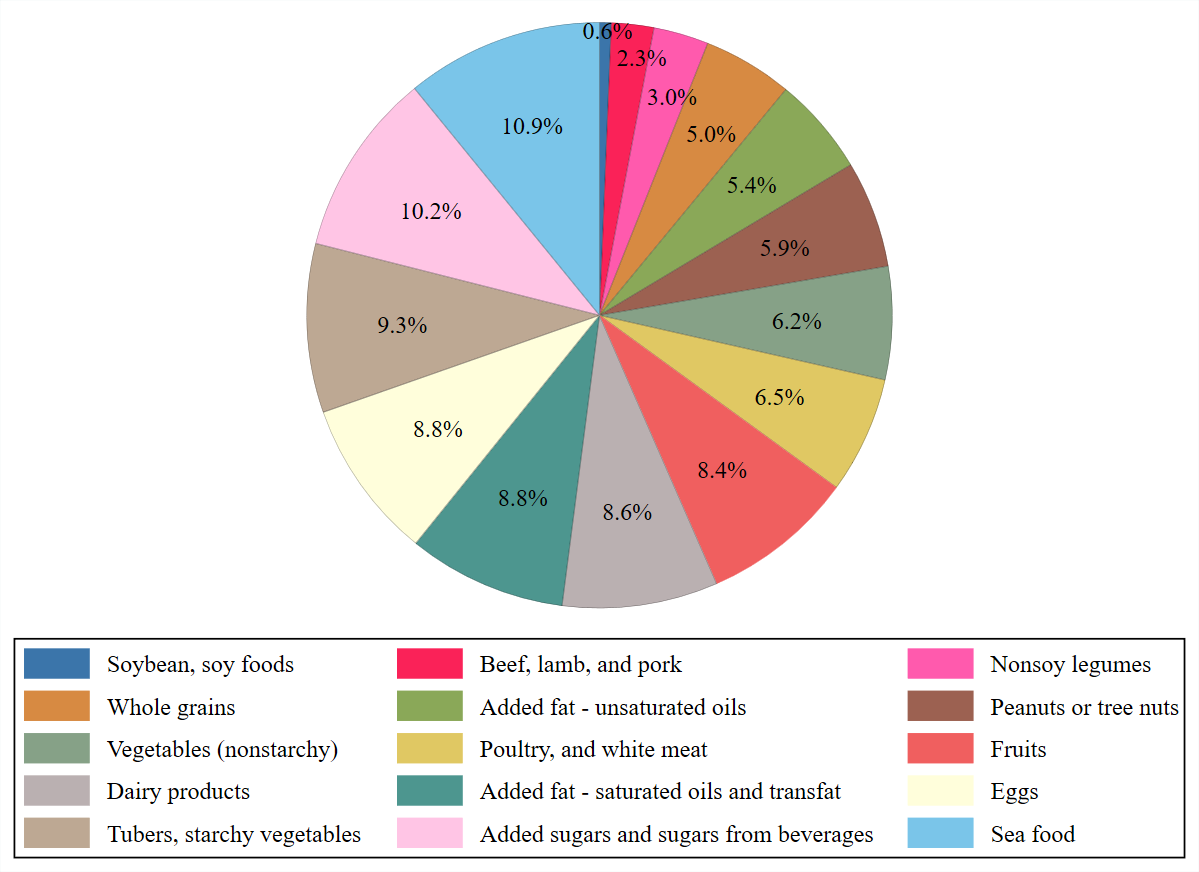


**Supplementary Figure 3.** Baseline adherence item proportion (%) of points within the PHDI (quartiles) score in the SI! Program, *n* = 886.

**
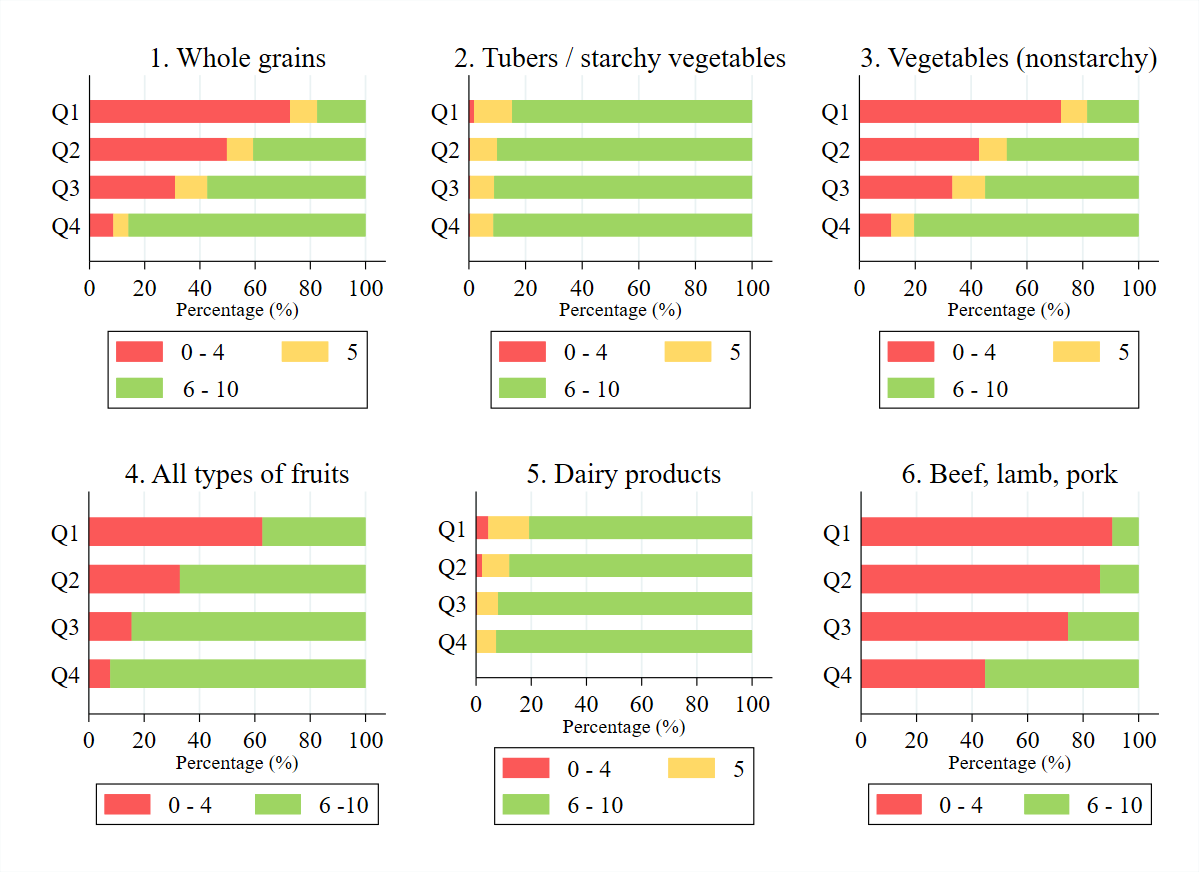
**

**
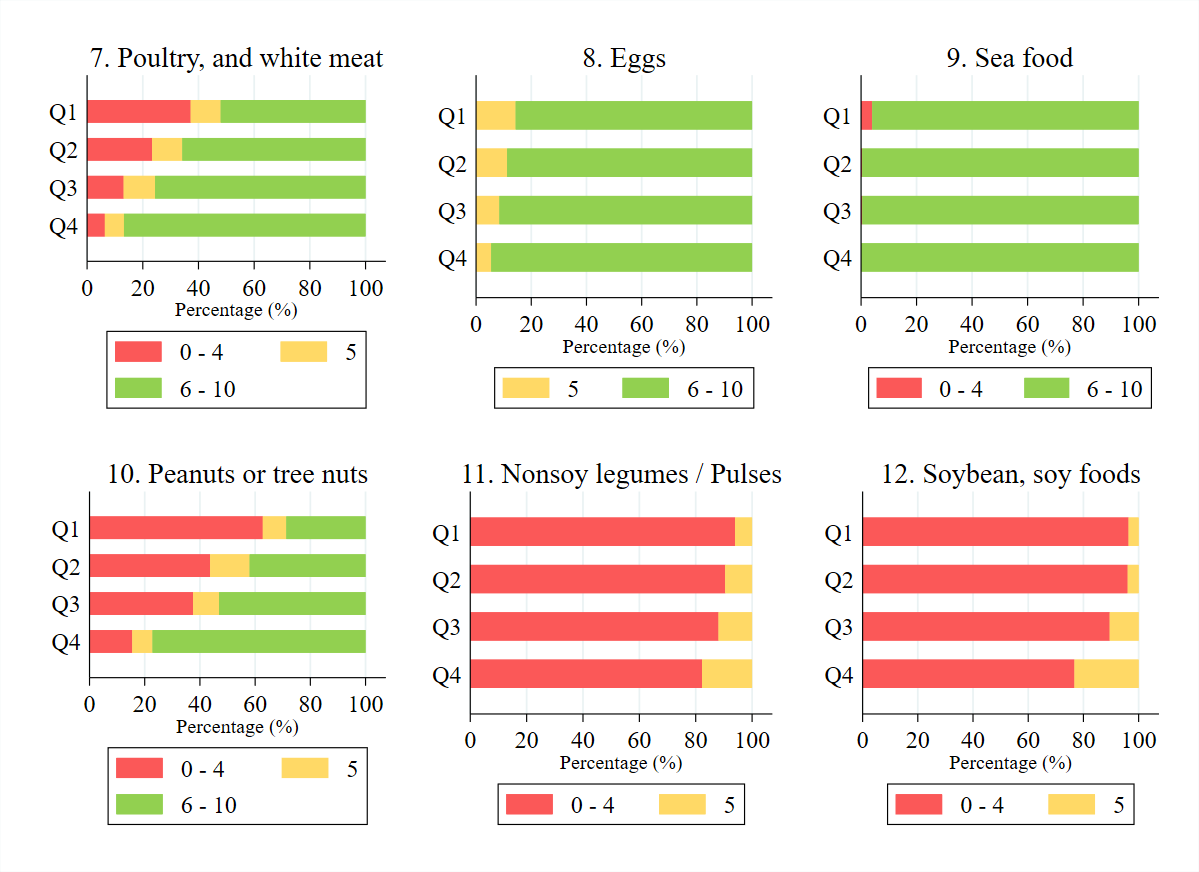
**

**
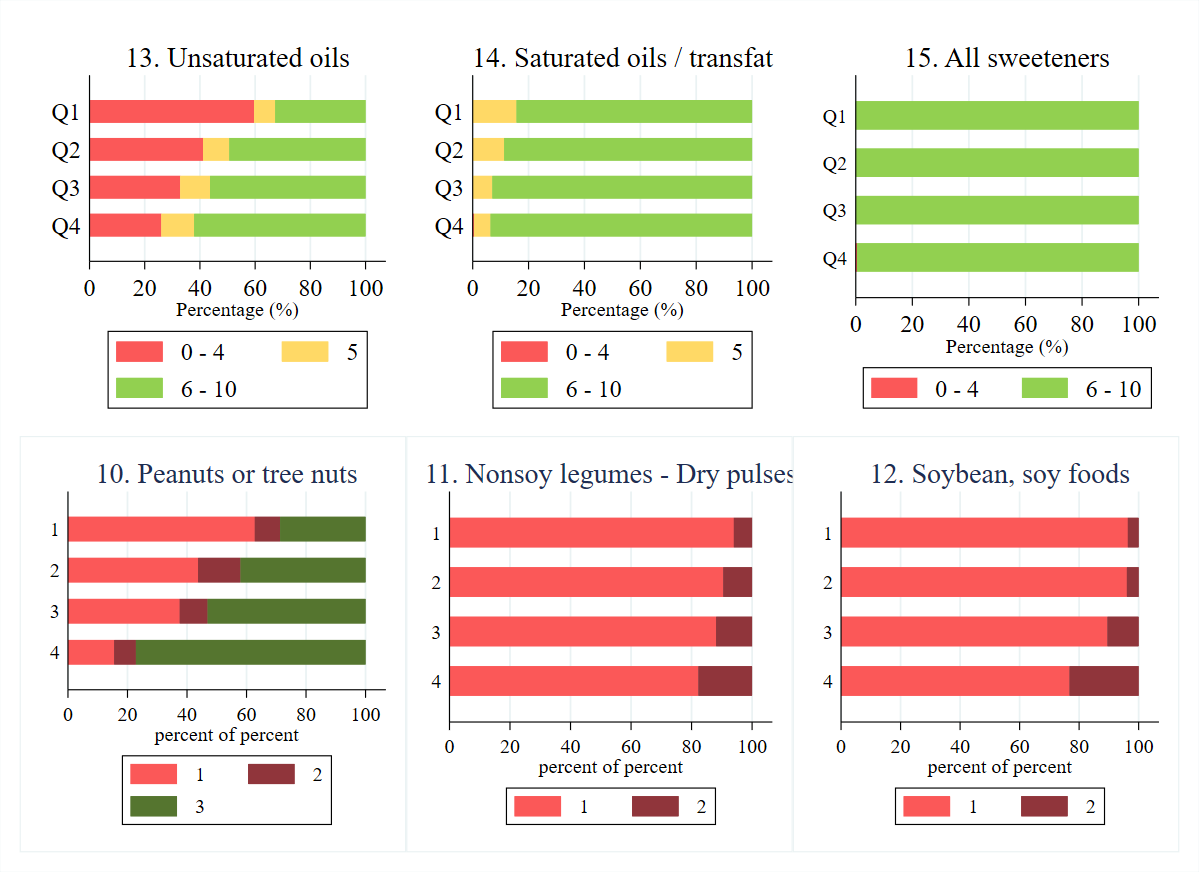
**

**Supplementary Figure 4.** Baseline adherence item proportion (%) of points within the PHDI (quartiles) score among migrant mothers (*n* = 163) in the SI! Program.

**
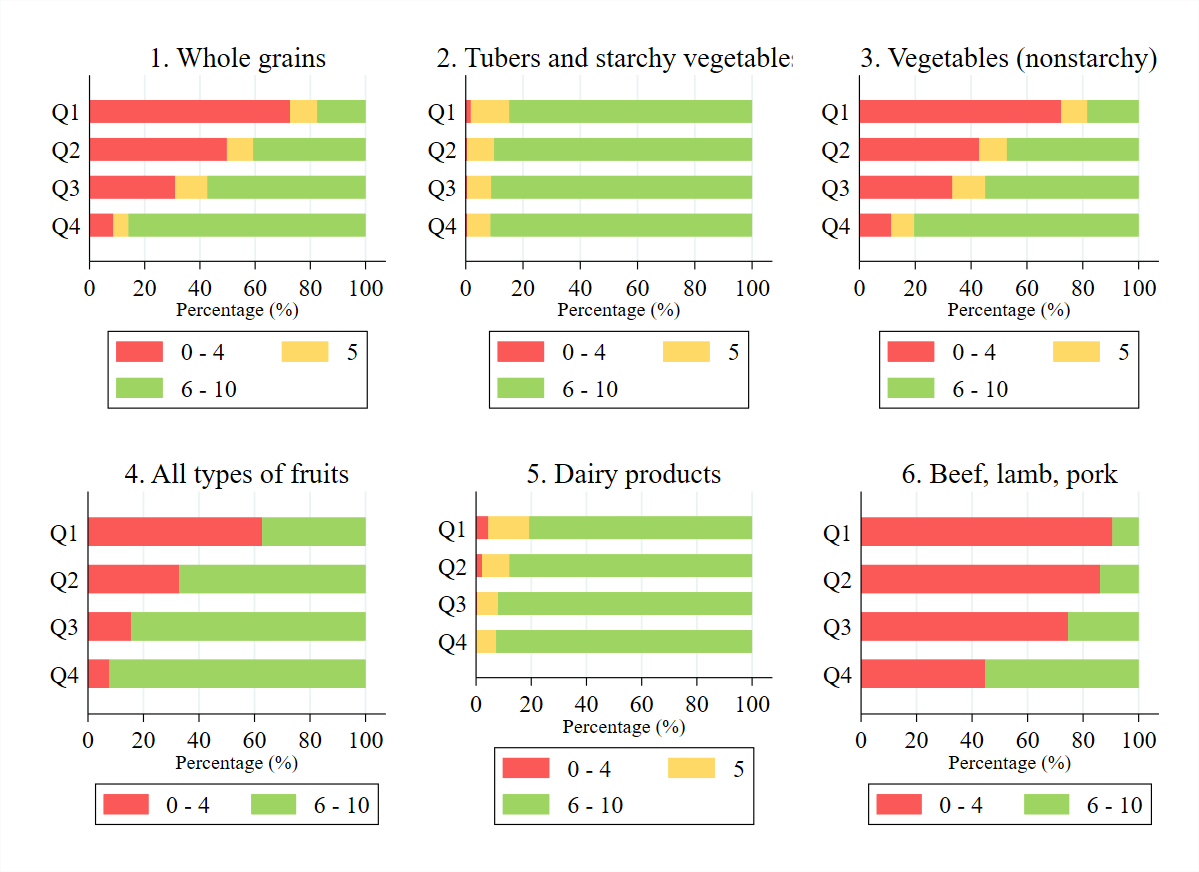

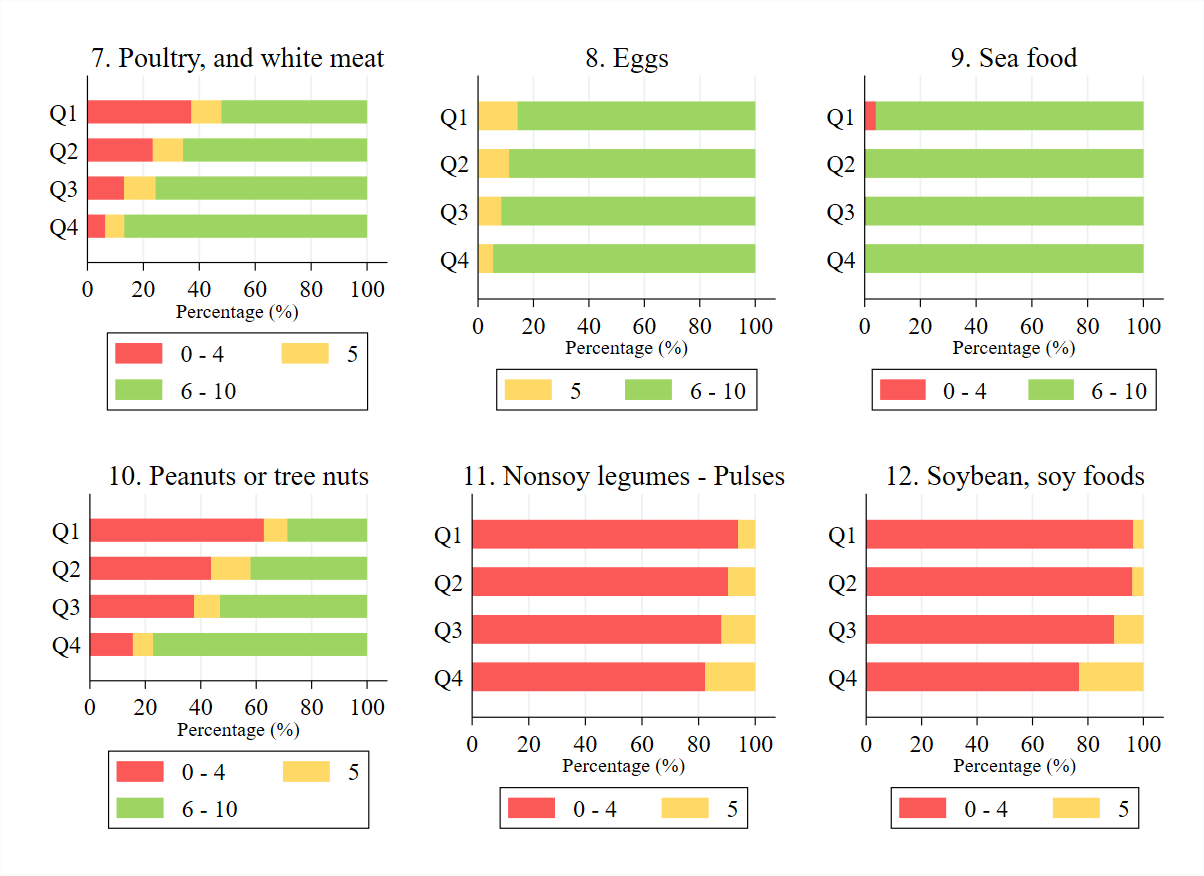
**

**
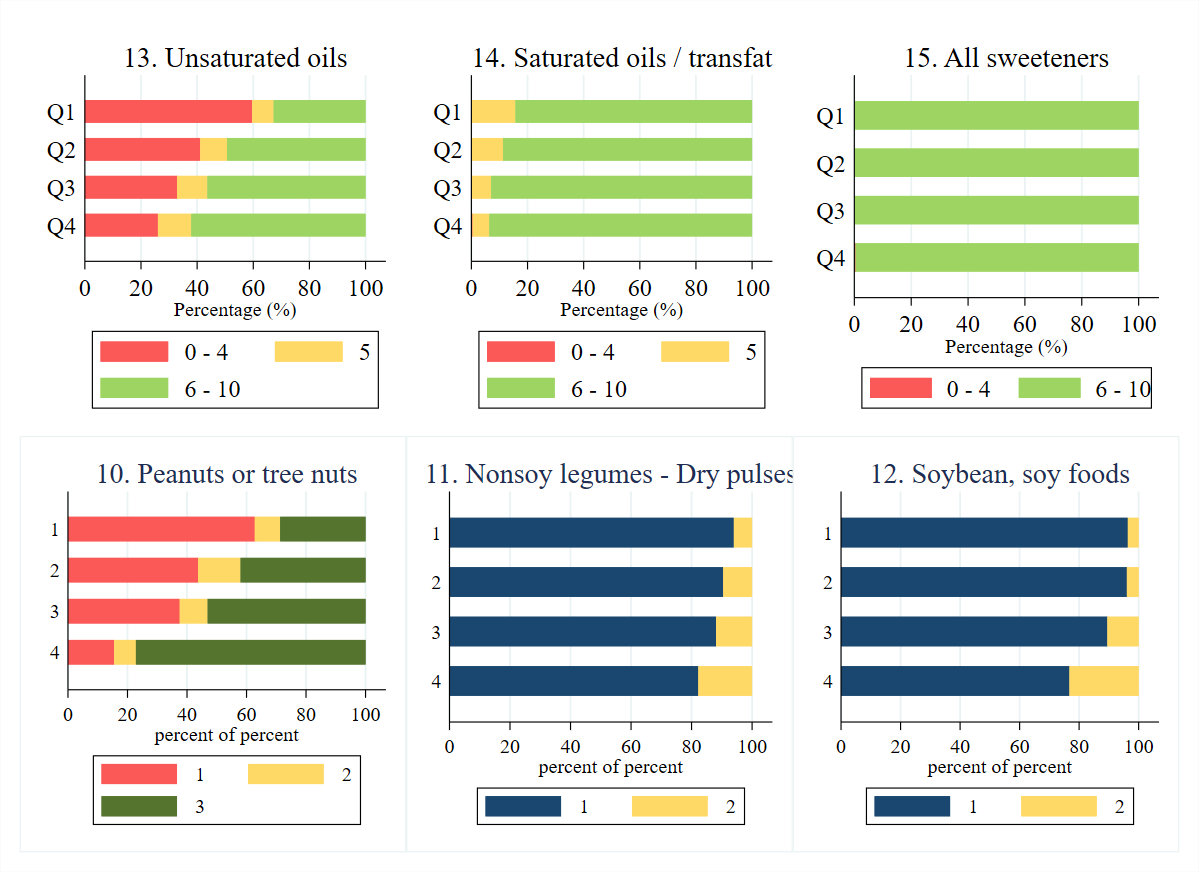
**

**Supplementary Figure 5.** Mean iron (mg/day), zinc (mg/day), vitamin B12, and calcium intake (mcg/day) by gender according to the PHDI in the SI! Program.


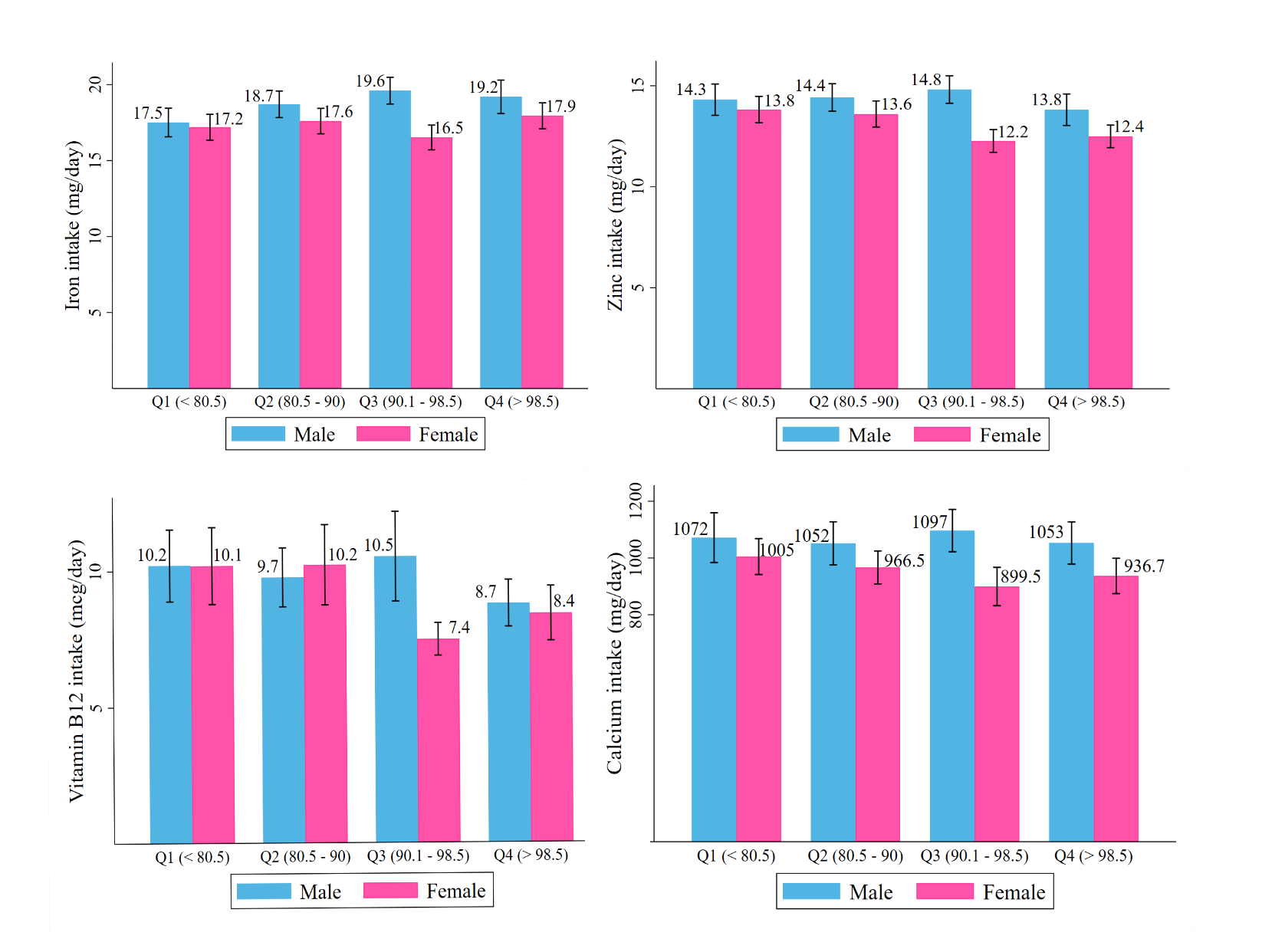


**Supplementary Figure 6.** Restricted cubic splines between the PHDI, and risk of new-onset high blood pressure and plasma cardiometabolic biomarkers, using Cox regression models (adjusted model B), in the SI! Program. Solid line is Hazard Ratios (HR), and dotted line is 95% CI.

**
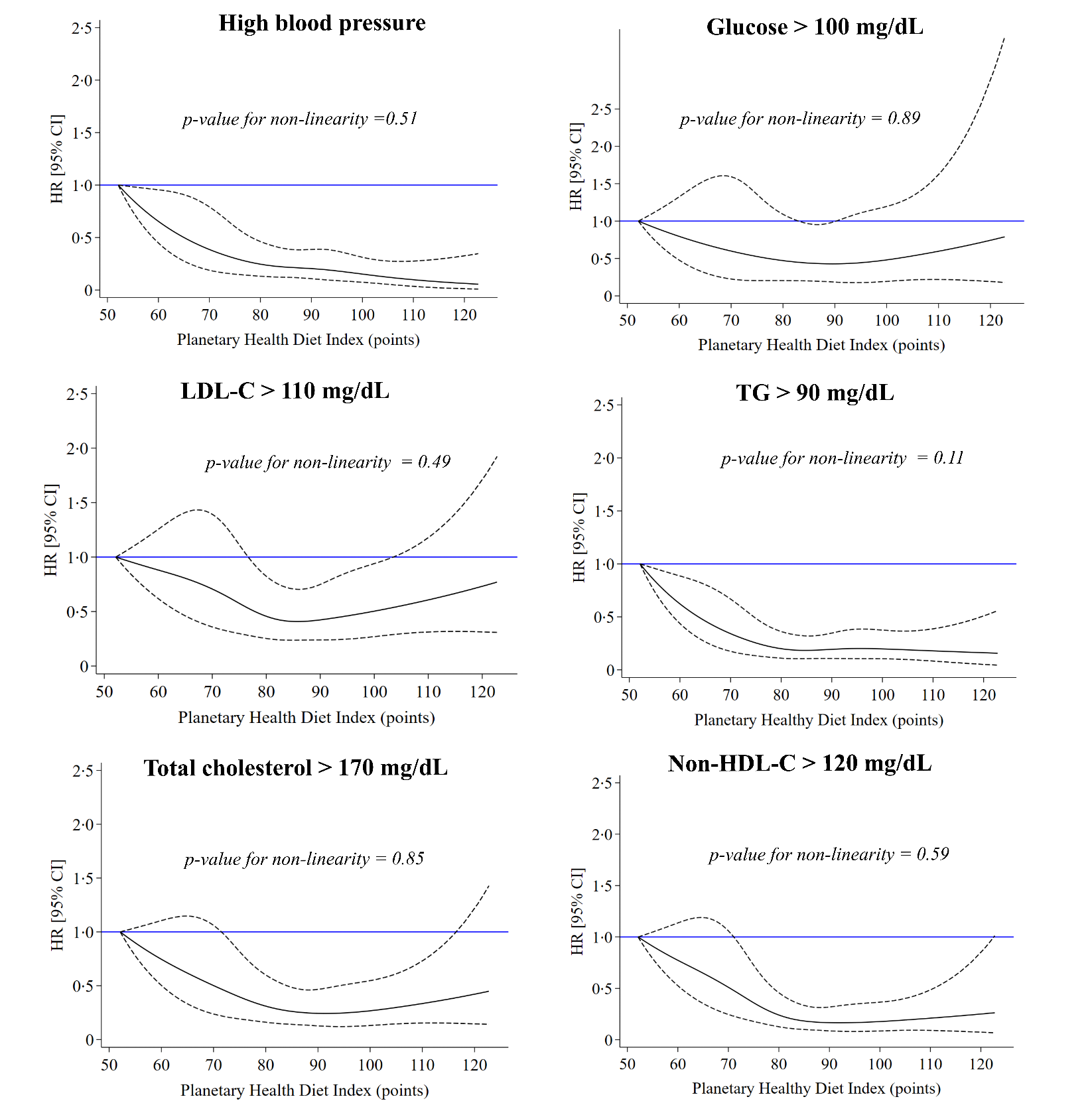
**

Abbreviations: CI: Confidence interval; HR: Hazard ratio; LDL-C: Low-density lipoprotein cholesterol; PHDI: Planetary Health Diet Index; TG: Triglycerides.

| **Supplementary Table 1.** Cox regression models^1^ for the PHDI, risk of new-onset high blood pressure, and elevated cardiometabolic biomarkers in the SI! Program, (no standardized diet). | | | | | | | | | | | | | | | | | | |  |
| --- | --- | --- | --- | --- | --- | --- | --- | --- | --- | --- | --- | --- | --- | --- | --- | --- | --- | --- | --- |
|  | **Q1** | **Q2** | | | | **Q3** | | | | **Q4** | | | |  |  | **Hazard ratio of PHDI for 20-point increase^2^** | | |  |
|  | **< 80.5** | **80.5 - 90** | | | | **90.1 - 98.5** | | | | **> 98.5** | | | |  |  |  |  |  |  |
| ***High blood pressure*** | |  |  |  |  |  |  |  |  |  |  |  |  |  |  |  |  |  |  |
| ***Cases/Person-years (219/2890)*** | 102 / 646 | 47 / 732 | | | | 41 / 756 | | | | 29 / 756 | | | |  |  |  |  |  |  |
| ***Incidence rate*** | 0.16 | 0.06 | | | | 0.05 | | | | 0.03 | | | |  |  |  |  |  |  |
|  |  | **HR** | **CI (95%)** | | ***p-value*** | **HR** | **CI (95%)** | | ***p-value*** | **HR** | **CI (95%)** | | ***p-value*** | ***p-trend*** |  | **HR** | **CI (95%)** | ***p-value*** |  |
| **Model A** | **Ref.** | 0.43 | 0.30 | 0.61 | **<0.001***** | 0.36 | 0.25 | 0.52 | **<0.001***** | 0.27 | 0.14 | 0.52 | **<0.001***** | **<0.001***** |  |  |  |  |  |
| **Model B** | **Ref.** | 0.46 | 0.34 | 0.64 | **<0.001***** | 0.36 | 0.27 | 0.49 | **<0.001***** | 0.25 | 0.15 | 0.40 | **<0.001***** | **<0.001***** |  | 0.44 | 0.34 0.56 | **<0.001***** |  |
|  |  |  |  |  |  |  |  |  |  |  |  |  |  |  |  |  |  |  |  |
| ***Glucose > 100 mg/dL*** | |  |  |  |  |  |  |  |  |  |  |  |  |  |  |  |  |  |  |
| ***Cases/Person-years (169/1020)*** | 57 / 220 | 41 / 314 | | | | 41 / 266 | | | | 30 / 220 | | | |  |  |  |  |  |  |
| ***Incidence rate*** | 0.26 | 0.13 | | | | 0.15 | | | | 0.14 | | | |  |  |  |  |  |  |
|  |  | **HR** | **CI (95%)** | | ***p-value*** | **HR** | **CI (95%)** | | ***p-value*** | **HR** | **CI (95%)** | | ***p-value*** | ***p-trend*** |  | **HR** | **CI (95%)** | ***p-value*** |  |
| **Model A** | **Ref.** | 0.58 | 0.54 | 0.62 | **<0.001***** | 0.61 | 0.50 | 0.76 | **<0.001***** | 0.59 | 0.40 | 0.86 | **<0.01**** | **<0.01**** |  |  |  |  |  |
| **Model B** | **Ref.** | 0.60 | 0.50 | 0.71 | **<0.001***** | 0.58 | 0.41 | 0.82 | **<0.01**** | 0.60 | 0.46 | 0.78 | **<0.001***** | **<0.01**** |  | 0.74 | 0.67 0.82 | **<0.001***** |  |
|  |  |  |  |  |  |  |  |  |  |  |  |  |  |  |  |  |  |  |  |
| ***LDL-C > 110 mg/dL*** | |  |  |  |  |  |  |  |  |  |  |  |  |  |  |  |  |  |  |
| ***Cases/Person-years (313/1966)*** | 103 / 456 | 78 / 494 | | | | 58 / 488 | | | | 75 / 526 | | | |  |  |  |  |  |  |
| ***Incidence rate*** | 0.23 | 0.16 | | | | 0.12 | | | | 0.15 | | | |  |  |  |  |  |  |
|  |  | **HR** | **CI (95%)** | | ***p-value*** | **HR** | **CI (95%)** | | ***p-value*** | **HR** | **CI (95%)** | | ***p-value*** | ***p-trend*** |  | **HR** | **CI (95%)** | ***p-value*** |  |
| **Model A** | **Ref.** | 0.69 | 0.51 | 0.94 | **0.020*** | 0.55 | 0.35 | 0.87 | **0.01**** | 0.67 | 0.34 | 1.33 | 0.26 | 0.26 |  |  |  |  |  |
| **Model B** | **Ref.** | 0.70 | 0.63 | 0.79 | **<0.001***** | 0.53 | 0.42 | 0.68 | **<0.001***** | 0.65 | 0.37 | 1.14 | 0.13 | 0.13 |  | 0.78 | 0.54 1.12 | 0.19 |  |
|  |  |  |  |  |  |  |  |  |  |  |  |  |  |  |  |  |  |  |  |
| ***TG > 90 mg/dL*** | |  |  |  |  |  |  |  |  |  |  |  |  |  |  |  |  |  |  |
| ***Cases/Person-years (242/2802)*** | 107 / 666 | 51 / 712 | | | | 41 / 696 | | | | 43 / 728 | | | |  |  |  |  |  |  |
| ***Incidence rate*** | 0.16 | 0.07 | | | | 0.05 | | | | 0.06 | | | |  |  |  |  |  |  |
|  |  | **HR** | **CI (95%)** | | ***p-value*** | **HR** | **CI (95%)** | | ***p-value*** | **HR** | **CI (95%)** | | ***p-value*** | ***p-trend*** |  | **HR** | **CI (95%)** | ***p-value*** |  |
| **Model A** | **Ref.** | 0.46 | 0.32 | 0.66 | **<0.001***** | 0.38 | 0.34 | 0.42 | **<0.001***** | 0.39 | 0.28 | 0.54 | **<0.001***** | **<0.001***** |  |  |  |  |  |
| **Model B** | **Ref.** | 0.48 | 0.35 | 0.65 | **<0.001***** | 0.37 | 0.34 | 0.40 | **<0.001***** | 0.39 | 0.28 | 0.55 | **<0.001***** | **<0.001***** |  | 0.53 | 0.42 0.68 | **<0.001***** |  |
| ***Total cholesterol > 170 mg/dL*** | | |  |  |  |  |  |  |  |  |  |  |  |  |  |  |  |  |  |
| ***Cases/Person-years (211/2136)*** | | 82 / 528 | 51 / 530 | | | | 40 / 504 | | | | 38 / 574 | | | |  |  |  |  |  |
| ***Incidence rate*** | | 0.16 | 0.10 | | | | 0.08 | | | | 0.07 | | | |  |  |  |  |  |
|  | |  | **HR** | **CI (95%)** | | ***p-value*** | **HR** | **CI (95%)** | | ***p-value*** | **HR** | **CI (95%)** | | ***p-value*** | ***p-trend*** |  | **HR** | **CI (95%)** | ***p-value*** |
| **Model A** | | **Ref.** | 0.64 | 0.49 | 0.83 | **<0.001***** | 0.53 | 0.36 | 0.79 | **<0.01**** | 0.42 | 0.24 | 0.76 | **<0.01**** | **<0.01**** |  |  |  |  |
| **Model B** | | **Ref.** | 0.69 | 0.60 | 0.81 | **<0.001***** | 0.53 | 0.44 | 0.65 | **<0.001***** | 0.42 | 0.30 | 0.59 | **<0.001***** | **<0.001***** |  | 0.62 | 0.50 0.78 | **<0.001***** |
| ***Non-HDL-C > 120 mg/dL*** | | |  |  |  |  |  |  |  |  |  |  |  |  |  |  |  |  |  |
| ***Cases/Person-years (202/2636)*** | | 96 / 632 | 46 / 664 | | | | 35 / 644 | | | | 25 / 696 | | | |  |  |  |  |  |
| ***Incidence rate*** | | 0.16 | 0.07 | | | | 0.05 | | | | 0.04 | | | |  |  |  |  |  |
|  | |  | **HR** | **CI (95%)** | | ***p-value*** | **HR** | **CI (95%)** | | ***p-value*** | **HR** | **CI (95%)** | | ***p-value*** | ***p-trend*** |  | **HR** | **CI (95%)** | ***p-value*** |
| **Model A** | | **Ref.** | 0.48 | 0.46 | 0.51 | **<0.001***** | 0.36 | 0.28 | 0.47 | **<0.001***** | 0.23 | 0.10 | 0.53 | **<0.001***** | **<0.001***** |  |  |  |  |
| **Model B** | | **Ref.** | 0.52 | 0.51 | 0.53 | **<0.001***** | 0.36 | 0.31 | 0.42 | **<0.001***** | 0.22 | 0.11 | 0.45 | **<0.001***** | **<0.001***** |  | 0.46 | 0.30 0.70 | **<0.001***** |
| Cox regression (clustering at recruitment municipality level and school) to conduct this analysis. Multivariable **model A**: gender (male / female), age (11-12 years / 13-14 years), parenteral education (primary / secondary / academic-graduate), randomized group (control / long-term intervention / short-term intervention), baseline Tanner maturation stage (from I to V). Multivariable **model B**: variables of model A plus, adolescent high blood pressure status (yes / no), adolescent BMI-for-age ( ≥ 5th to < 85th percentile / ≥ 85th to < 95th percentile/ ≥ 95th percentile), moderate to vigorous physical activity 60 min-day (yes / no), sleep duration (hours, continuous), energy intake (kcal/day, continuous). For high blood pressure analysis the model B further included: dietary sodium and potassium ratio (continuous), and calcium (mg/day, continuous); both adjusted for total energy using the residual method.  P-value <0.05 considered significant, values shown in bold are statistically significant (*p ≤ 0.05; **p ≤ 0.01; ***p ≤ 0.001).  ^1^ Number of participants not having the condition or the elevated cardiovascular marker at baseline: High BP: *n* = 769; Glucose: *n* = 320; LDL-C: 597; TG: *n* = 509; Total cholesterol: *n* = 856; Non-HDL-C: *n* = 719 | | | | | | | | | | | | | | | | | | |  |
| ^2^ Fitted according to model B. |  |  |  |  |  |  |  |  |  |  |  |  |  |  |  |  |  |  |  |
| Abbreviations: CI: Confidence interval; HR: Hazard ratio; LDL-C: Low-density lipoprotein cholesterol; PHDI: Planetary Health Diet Index; TG: Triglycerides. | | | | | | | | | | | | | | | | | | |  |

**Supplementary Table 2.** STROBE Statement—checklist of items that should be included in reports of observational studies

|  | **Item No** | **Recommendation** | **Page  No** |
| --- | --- | --- | --- |
| **Title and abstract** | 1 | (*a*) Indicate the study’s design with a commonly used term in the title or the abstract | 1 |
|  |  | (*b*) Provide in the abstract an informative and balanced summary of what was done and what was found | 2 |
| **Introduction** | | | |
| Background/rationale | 2 | Explain the scientific background and rationale for the investigation being reported | 3-4 |
| Objectives | 3 | State specific objectives, including any prespecified hypotheses | 3-4 |
| **Methods** | | | |
| Study design | 4 | Present key elements of study design early in the paper | 4 |
| Setting | 5 | Describe the setting, locations, and relevant dates, including periods of recruitment, exposure, follow-up, and data collection | 4 |
| Participants | 6 | (*a*) *Cohort study*—Give the eligibility criteria, and the sources and methods of selection of participants. Describe methods of follow-up  *Case-control study*—Give the eligibility criteria, and the sources and methods of case ascertainment and control selection. Give the rationale for the choice of cases and controls  *Cross-sectional study*—Give the eligibility criteria, and the sources and methods of selection of participants | 4  -  - |
|  |  | (*b*) *Cohort study*—For matched studies, give matching criteria and number of exposed and unexposed  *Case-control study*—For matched studies, give matching criteria and the number of controls per case | -  - |
| Variables | 7 | Clearly define all outcomes, exposures, predictors, potential confounders, and effect modifiers. Give diagnostic criteria, if applicable | 6-7 |
| Data sources/ measurement | 8* | For each variable of interest, give sources of data and details of methods of assessment (measurement). Describe comparability of assessment methods if there is more than one group | 5-6 |
| Bias | 9 | Describe any efforts to address potential sources of bias | 5, 7 |
| Study size | 10 | Explain how the study size was arrived at | 4 |
| Quantitative variables | 11 | Explain how quantitative variables were handled in the analyses. If applicable, describe which groupings were chosen and why | 6-7 |
| Statistical methods | 12 | (*a*) Describe all statistical methods, including those used to control for confounding | 6-7 |
|  |  | (*b*) Describe any methods used to examine subgroups and interactions | 6 |
|  |  | (*c*) Explain how missing data were addressed | 6 |
|  |  | (*d*) *Cohort study*—If applicable, explain how loss to follow-up was addressed  *Case-control study*—If applicable, explain how matching of cases and controls was addressed  *Cross-sectional study*—If applicable, describe analytical methods taking account of sampling strategy | 6  -  - |
|  |  | (*e*) Describe any sensitivity analyses | 6 |

| **Results** | | | |
| --- | --- | --- | --- |
| Participants | 13* | (a) Report numbers of individuals at each stage of study—eg numbers potentially eligible, examined for eligibility, confirmed eligible, included in the study, completing follow-up, and analysed | 6 |
|  |  | (b) Give reasons for non-participation at each stage | - |
|  |  | (c) Consider use of a flow diagram | Figure 1 |
| Descriptive data | 14* | (a) Give characteristics of study participants (eg demographic, clinical, social) and information on exposures and potential confounders | 7, Table 1-2, Supplementary Figure 1-5 |
|  |  | (b) Indicate number of participants with missing data for each variable of interest | 6 |
|  |  | (c) *Cohort study*—Summarise follow-up time (eg, average and total amount) | 8 |
| Outcome data | 15* | *Cohort study*—Report numbers of outcome events or summary measures over time | 8, Table 3-4 |
|  |  | *Case-control study—*Report numbers in each exposure category, or summary measures of exposure | *-* |
|  |  | *Cross-sectional study—*Report numbers of outcome events or summary measures | *-* |
| Main results | 16 | (*a*) Give unadjusted estimates and, if applicable, confounder-adjusted estimates and their precision (eg, 95% confidence interval). Make clear which confounders were adjusted for and why they were included | 7-9, Table 3-4 |
|  |  | (*b*) Report category boundaries when continuous variables were categorized | 5 |
|  |  | (*c*) If relevant, consider translating estimates of relative risk into absolute risk for a meaningful time period | - |
| Other analyses | 17 | Report other analyses done—eg analyses of subgroups and interactions, and sensitivity analyses | Figure 2, Supplementary Table 1,  Supplementary Figure 6. |
| **Discussion** | | | |
| Key results | 18 | Summarise key results with reference to study objectives | 9-10 |
| Limitations | 19 | Discuss limitations of the study, taking into account sources of potential bias or imprecision. Discuss both direction and magnitude of any potential bias | 11 |
| Interpretation | 20 | Give a cautious overall interpretation of results considering objectives, limitations, multiplicity of analyses, results from similar studies, and other relevant evidence | 9-10 |
| Generalisability | 21 | Discuss the generalisability (external validity) of the study results | 9-10 |
| **Other information** | | | |
| Funding | 22 | Give the source of funding and the role of the funders for the present study and, if applicable, for the original study on which the present article is based | 12 |
